# Learning with others: teacher–learner brain synchrony depends on mutual gaze and joint attention

**DOI:** 10.1093/cercor/bhaf323

**Published:** 2025-12-01

**Authors:** Sara De Felice, Francesco Di Ciò, Danny Tompkins, Uzair Hakim, Paola Pinti, Gabriella Vigliocco, Antonia F de C Hamilton

**Affiliations:** Department of Psychology, University of Cambridge, Old Cavendish Building, Rayleigh Wing, Free School Lane, CB2 3RF, Cambridge, United Kingdom; Institute of Cognitive Neuroscience, University College London, 17 Queen Square, WC1N 3AZ, London, United Kingdom; Institute of Cognitive Neuroscience, University College London, 17 Queen Square, WC1N 3AZ, London, United Kingdom; Institute of Cognitive Neuroscience, University College London, 17 Queen Square, WC1N 3AZ, London, United Kingdom; Department of Clinical, Educational and Health Psychology, University College London, 1-19 Torrington Place, WC1E 7HB, London, United Kingdom; Department of Medical Physics and Biomedical Engineering, University College London, Malet Place Engineering, Gower St, WC1E 6BT, London, United Kingdom; Centre for Brain and Cognitive Development, Department of Psychological Sciences, Birbeck, University of London, Henry Wellcome Building, 32 Torrington Square, WC1E 7JL, London, United Kingdom; Psychology and Language Science, University College London, 26 Bedford Way, WC1H 0AP, London, United Kingdom; Institute of Cognitive Neuroscience, University College London, 17 Queen Square, WC1N 3AZ, London, United Kingdom

**Keywords:** brain synchrony, hyperscanning, joint attention, relational neuroscience, social learning

## Abstract

Successful learning often emerges through social interaction: what are the neural and behavioral systems that support this process? This ecological, multimodal study combines functional near-infrared spectroscopy hyperscanning with detailed behavioral and physiological measures in 27 unconstrained social learning interactions. Learning was supported by teacher–learner interbrain synchrony (interpersonal neural synchrony), over regions important for mutual understanding (temporoparietal junction) and communicative coordination (ventral premotor cortex). Joint attention and mutual gaze modulated the interpersonal neural synchrony–learning association in oppositive ways, motivating a dual-process model: during knowledge-building phases, learning is supported by informational uptake dynamic, with high joint attention, low interpersonal neural synchrony in regions for mutual understanding (temporoparietal junction) and coordination (right ventral premotor cortex), and high interpersonal neural synchrony in language-related areas (left ventral premotor cortex). In contrast, during moments of mutual grounding, learning is supported by high mutual gaze and high interpersonal neural synchrony over temporoparietal junction. Cross-brain general linear modeling revealed asymmetric neural dependencies linked to speaking and teaching roles in the left-hemisphere language network. These effects remained after controlling for nodding, gaze, and breathing, indicating that interpersonal neural synchrony reflects true social-cognitive alignment beyond sensorimotor coupling. Taken together, this study shows that successful learning arises from coordinated and nonlinear brain–body dynamics and positions interpersonal neural synchrony as a marker of mutual prediction during communicative social interaction.

## Introduction

The ability to teach and learn new information in interaction with others is a crucial human skill that supports knowledge-sharing and underpins cultural development. Accumulating evidence suggests that learning in social interaction outperforms noninteractive learning (Kuhl et al. 2003; Lytle et al. 2018; Kostyrka-Allchorne et al. 2019; De Felice et al. 2021, 2022, 2023). However, the neurophysiological and cognitive underpinnings of this phenomenon remain elusive. Interpersonal neural synchrony (INS), commonly measured as coherence across multiple brains (Hakim et al. 2023), has been identified as a neural correlate of social learning (Liu et al. 2019; Shamay-Tsoory 2022; Pan et al. 2023; Davidesco et al. 2023). Interpersonal behavioral dynamics, including shared attention and eye-gaze coordination, have been linked to both INS (Leong et al. 2017; Piazza et al. 2021; Wohltjen and Wheatley 2021; Rosen et al. 2025) and learning (Mundy and Newell 2007; Ishikawa and Yoshioka 2025). Yet, a detailed neurocognitive model that takes into account both brain and behavior in interactive social learning remains unavailable. This study aims to explicate the relationships between INS, interactive behaviors, and learning in adult humans.

### Interactive learning and the brain

Learning new information often takes place in the context of social interaction, for example in interaction with caregivers during infancy and childhood, and from teachers, peers, and colleagues throughout our life. With the advent of neuroimaging modalities suitable to record data in real-world settings, including functional near-infrared spectroscopy (fNIRS) and electroencephalography (EEG), an increasing number of studies are exploring how brain activity patterns relate to learning in naturalistic and interactive contexts. Hyperscanning methods record brain activity from two or more participants simultaneously, allowing researchers to quantify the INS between two people (Babiloni and Astolfi 2014; Nam et al. 2020; Hakim et al. 2023). INS is a measure of how similar brain activity patterns are across time, and it has been linked to a wide range of relational dynamics (De Felice et al. 2025), including teacher–learner interactions (Reindl et al. 2024).

A growing body of literature suggests that teacher–learner INS may be linked to successful learning (eg Bevilacqua et al. 2019; Davidesco et al. 2023; Dikker et al. 2017; Pan et al. 2020a, 2021, 2023). For example, in a five-person EEG hyperscanning study, Davidesco et al. (2019) simultaneously measured brain activity from four students and their teacher during a science class. They found that alpha-band brain-to-brain coherence predicted students’ learning, as measured via performance in an immediate and a delayed test a week after the class. Moreover, moment-to-moment variation in alpha-band brain-to-brain coherence during the class specifically predicted what information was retained by the students a week later. Using fNIRS, Holper et al. (2013) recorded prefrontal brain activity in 17 teacher–student pairs and found that learning—as measured by students’ correct responses—was associated with higher correlation of student–teacher brain activity. Similar findings were obtained by another group who also used fNIRS to measure brain activity from learner and teacher dyads during the acquisition of a music song (Pan et al. 2018). They found that brain activity in the bilateral inferior frontal cortex showed learner–teacher synchronization. This was specifically associated with moments when the learner was observing the teacher and when learning was more interactive (measured in terms of turn-taking). Importantly, learner–teacher brain synchronization could predict student’s performance on the learned song.

Overall, these studies demonstrate that INS during interactive learning is positively associated with learning performance. However, a major challenge in INS research is the appropriate interpretation of these neural synchrony measures. INS cannot emerge spontaneously between brains; rather, it is likely shaped by the sensory and motor signals exchanged between individuals during interaction. INS may reflect mutual prediction (Hamilton 2021; Mayo and Shamay-Tsoory 2024), whereby one individual anticipates the actions of their partner, resulting in shared neural activation. Evidence of these effects have been reported in single-cell recordings from mice (Kingsbury et al. 2019) and in cortical activity from humans (Cañigueral et al. 2021). Conceptual alignment during conversation, such as shared understanding and consensus, may also give rise to INS (Sievers et al. 2024). Central to these accounts is the idea that the behavioral dynamics of interaction—ie patterns of gaze, speech, and movement—are key drivers of INS. Accordingly, a detailed examination of behavior is essential for elucidating the neural mechanisms underlying INS.

### Social behavior and learning

The role of behavior has been acknowledged in studies of social learning. For example, the INS–learning association has been proposed to be mediated by shared attention (Dikker et al. 2017), turn-taking (Holper et al. 2013), and/or motor coordination (Pan et al. 2018, 2020b). Pan et al. (2018, 2020b) used video-based analyses to capture interactional behaviors such as turn-taking, observation of the instructor, and spontaneous body-movement synchrony. These coded behaviors were aligned with interbrain synchrony measures and shown to predict learning of a song. However, previous studies are limited in that they tend to focus on one behavioral variable only, and this variable is often tightly bound to the content being learned (eg imitation of singing in song-learning tasks) rather than reflecting broader communicative dynamics. Overall, the challenges posed by collecting and analyzing complex multimodal dataset has led many social learning models to overlook behavioral measures. In the present study, we take a multimodal approach to communicative behaviors, examining how different factors support the learning of factual knowledge in the context of face-to-face naturalistic social interaction. Specifically, we aim to integrate brain and behavioral recordings (Jones et al. 2025) in order to understand what factors can predict successful interactive learning and why.

Previous work has identified some key behavioral components of social interaction, including eye-gaze (namely, joint attention and mutual gaze; Cañigueral et al. 2021; Degutyte and Astell 2021; Eilan et al. 2005; Mundy and Newell 2007; Saito et al. 2010; Wohltjen and Wheatley 2021), nodding (Thepsoonthorn et al. 2016; Danner et al. 2021), and speech (Garrod and Pickering 2004; Richardson et al. 2007; Kawasaki et al. 2013; Nguyen et al. 2021). Note that learning with others can be viewed as a specific form of social interaction, characterized by explicit teacher–learner roles and a clear learning goal. Rather than relying on specific mechanisms, teacher and learner most likely engage the same communicative mechanisms that support any smooth interactions (eg turn-taking, joint attention, mutual gaze, and nodding). These communicative mechanisms may be coordinated in specific ways that serve the particular demands of learning and knowledge transfer. They form the focus of our investigation and are briefly reviewed below. In addition, we include breathing primarily to control for physiological noise in fNIRS data (Tachtsidis and Scholkmann 2016), although we acknowledge that it may also provide insight into co-regulation during social interaction (Kaiser and Butler 2021; Balconi et al. 2023; Konvalinka et al. 2023).

*Eye-gaze.* Recently, we demonstrated that adults who learn in a videocall with a live teacher could retain more information compared to when they learned from a recorded video and that seeing the full face of the teacher (including their eyes) during the interaction was associated with the highest learning (De Felice et al. 2021). Consistent with our findings, previous work has identified gaze as an important social signal during communication (Richardson and Dale 2005; Richardson et al. 2007; Wohltjen and Wheatley 2021). Gaze is considered one of the strongest factors driving brain synchrony (Saito et al. 2010; Dumas et al. 2011; Kelsen et al. 2020; Noah et al. 2020). This may be because eye-gaze behavior serves as a cognitive proxy for attention: when teacher and learner focus their gaze to the same object of interest, they engage in *joint attention*, ie moments of shared thinking and co-construction of common ground. Infants as young as 6 months are sensitive to joint attention as a communicative cue (Senju and Csibra 2008), and joint attention is a key predictor of learning in early childhood (Elsabbagh et al. 2012; Ho et al. 2015; Yu et al. 2017; Lanthier et al. 2021; Piazza et al. 2021). In adults, joint attention has been linked to class engagement (Alksne 2016), effective communication (Kourtis et al. 2020; Wohltjen and Wheatley 2021), and learning (Mundy and Newell 2007). Mutual gaze has been studied less often, but one paper suggests that it may play a corrective role in disrupting shared attention to facilitate independent contributions to conversation (Wohltjen and Wheatley 2021).

*Nodding.* Head nodding represents a distinct social signal typical of naturalistic conversations (Kendon 2002; Poggi et al. 2010). This can reflect different functions, from signaling attention and understanding (Thepsoonthorn et al. 2016), to coordinate speech exchanges and passing turns (Danner et al. 2021). Previous research showed that in structured conversations, participants produce fast nods as they listen to new information (Hale et al. 2020), suggesting that nodding may be a backchannel in dialogues. This may be particularly relevant in learner–teacher interactive scenarios. For example, it has been suggested that nodding behavior change has a function of prior knowledge about a given topic (Thepsoonthorn et al. 2016). In the case of learner–teacher interaction, nodding may therefore be used by the learner to feedback understanding and signal to the teacher that they can move on and share further information. Note that the frequency, kinematics, and meaning of head nods are culture dependent (Matsumoto and Hwang 2013): our sample consisted of participants based in London, United Kingdom, and interpretations should therefore be considered within this cultural context.

*Speech.* Dialogue is a hallmark of interactive learning, which outperforms noninteractive learning such as prerecorded video watching (De Felice et al. 2021, 2023). Speech—especially through verbal turn-taking—has been associated with both INS and successful learning. For example, Nguyen and colleagues showed that mother–child INS over frontal and temporoparietal brain regions was associated with high frequency of vocal turn-taking (Nguyen et al. 2021). The same group showed that turn-taking was associated with INS between mothers and their babies as young as 4 months, and was predictive of later vocabulary size (Nguyen et al. 2023). In adults, INS has been associated with turn-taking behavior during conversations (Hirsch et al. 2018), with higher INS in face-to-face dialogues compared to monologues (Jiang et al. 2012).

*Breathing.* Breathing was included primarily to control for physiological noise in fNIRS data since systemic physiology can strongly influence measured signals (Tachtsidis and Scholkmann 2016). Beyond this methodological role, we acknowledge that breathing is increasingly recognized as a key component of naturalistic social interaction, possibly reflecting mechanisms of co-regulation between interlocutors (Tschacher and Meier 2020; De Felice et al. 2025). Although such synchrony may partly reflect predictable physiological patterns rather than reciprocity per se (Konvalinka et al. 2023), it highlights the possible relevance of breathing to moments of alignment during social learning.

### The current study

The short review above illustrates that there are at least three distinct factors to consider in exploring the neuroscience of social interactive learning: neural activation, social behavior, and learning success. Prior work has often focused on neural measures alone, overlooking the role of behavior in shaping social learning. We propose that social behavior should be regarded as a core component of social learning models, interacting with neural activation in ways that characterize pedagogical exchanges. Previous work suggests that several plausible models can be hypothesized: neural synchrony may influence behaviors that support learning (Pan et al. 2020b; Yang et al. 2021); synchrony may instead arise from specific social behaviors which then facilitate learning (Hamilton 2021); or a common factor such as engagement or motivation may drive both. While the current study does not aim to resolve questions of directionality, its contribution lies in positioning behavior as an integral part of the neuroscience of social learning and demonstrating how behavioral and neural processes jointly relate to learning outcomes.

**Fig. 1:**
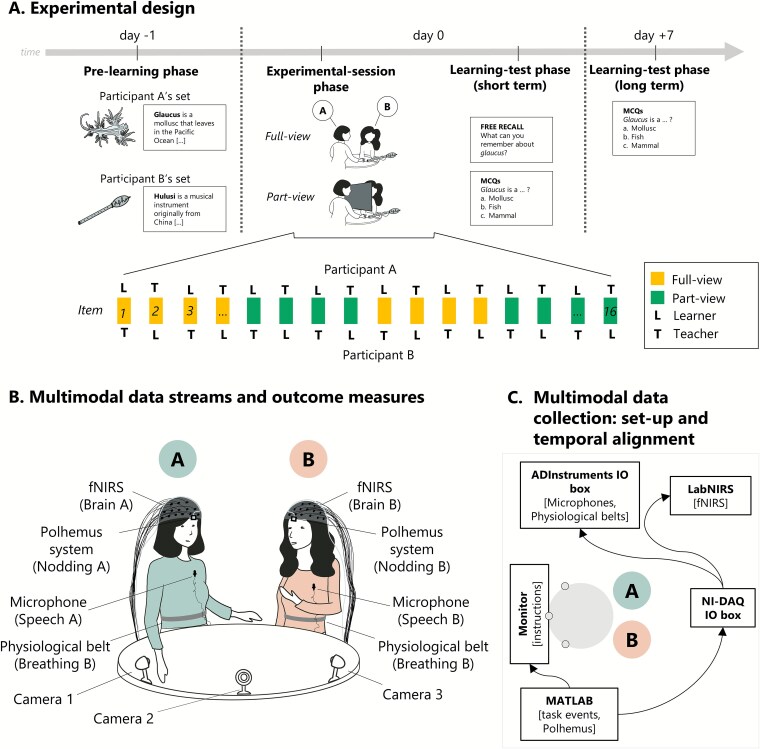
Methods. A. Experimental design pre-learning phase (day −1): Participants are asked to learn, in their own time, 8 items each. This phase occurs at home before coming to the laboratory. Only once participant reaches 100% of learning of these items they are invited to the experimental session. Experimental session and short-term learning phase (day 0): Participants complete the social learning task in the laboratory, where they teach 8 items and learn about other 8 items from their partner. Their learning is tested immediately after the interaction. Long-term learning-test phase (day +7): After 1 week, participants’ learning is tested again via a multiple-choice quiz online. Note that both short-term and long-term learning tests focus exclusively on the items learned socially during the experimental session (day 0). B. Multimodal data streams and outcome measures schematic of devices, data streams, and respective outcome measures collected during the experimental-session phase. C. Multimodal data collection: Set-up and temporal alignment top-down view of experimental-session room. Data streams were recorded through different devices (see also Table S1): Nodding was recorded using the Polhemus liberty system and processed in MATLAB; speech and breathing data were recorded using breathing belts and an ADInstruments data acquisition box and processed through LabChart software, and brain data were recorded via the Shimadzu LabNIRS system. MATLAB software also recorded task events and sent timing triggers to both ADInstruments IO box and LabNIRS through NI-DAQ IO box.

In our study, we use fNIRS hyperscanning to simultaneously measure brain activity from two individuals engaged in a highly naturalistic learner–teacher interaction. We employed fNIRS, the most widely used modality for hyperscanning (Hakim et al. 2023), as it captures slower changes in brain state aligned with the time course of understanding in conversation (Hamilton et al. 2025) and is robust to the head and eye movements typical of naturalistic teacher–learner settings. Prior to the laboratory session, participants independently learn facts about obscure items (eg exotic animals). In the laboratory, they take turns teaching and learning these facts with a partner in free-form conversations. Alongside neural data, we collect detailed behavioral measures—including gaze, speech, nodding, and breathing—and assess learning outcomes both immediately and after a delay. To manipulate the richness of social interaction, we include trials where participants can see each other fully and others where a barrier blocks their partner’s face (see Fig.  1A). The primary data analyzed in this study include (1) brain activity in regions of interest, (2) second-by-second coding of joint attention and mutual gaze per trial, (3) second-by-second coding of whether each participant is speaking or listening, and (4) learning scores for each item taught per trial.

There are at least two ways to examine this type of complex data. One option is to ask which factors predict successful learning. Here, we start from the brain data from each trial in terms of the average wavelet coherence in specific frequency bands (Cui et al. 2011; Hakim et al. 2023), a widely used measure of INS. We then assess how INS, alongside behavioral measures, predicts learning outcomes across trials and participants. Based on prior fNIRS studies of social communication (Cañigueral et al. 2021; Fronda and Balconi 2020; Jiang et al. 2015), we hypothesize that INS in brain networks involved in social cognition and communication—especially TPJ—will be associated with better learning. We also hypothesize, in line with studies showing the role of joint attention in effective communication and information sharing (Richardson et al. 2007; Kourtis et al. 2020), that joint attention would be positively associated with learning. Given the limited number of studies examining mutual gaze, and the scarcity of research integrating eye-gaze behavior with interbrain synchrony (INS) to predict learning outcomes, we do not make strong predictions regarding mutual gaze or the interaction between gaze behaviors and INS in supporting learning.

A second approach to examining this complex dataset it to take an embodied social neuroscience approach (Kingsbury and Hong 2020; Hamilton 2021) to explore what factors drive brain activity in a single participant. Here, we leave aside the learning performance and ask how single measures of behavior (speech, nodding, and eye-gaze) recorded at each time point, physiology (breathing), and partner’s brain activity (fNIRS data) can predict an individual participant’s brain activity. This cross-brain general linear model (xGLM) approach has the potential to give more detailed insights into the specific relationships between behavior and brain activity in the context of naturalistic interaction (Cañigueral et al. 2021) and to reveal if mutual prediction is taking place that goes beyond effects driven by observable behavior. Here, we will build a series of models using gaze behavior, speech, nodding, physiology, and partner’s brain activity to predict patterns of activation in each individual. Because these models consider one participant at a time, we can explore asymmetric behaviors (eg speaking/listening) and asymmetric roles (teaching/learning) as well as the symmetric behaviors (joint attention) tested in the first analysis. We predict that different neural systems may be revealed by this asymmetric analysis, with particular effects of speech in the left hemisphere.

In summary, this study examines the triadic relationship between behavior, brain activity, and successful learning in naturalistic teacher–learner interactions. Specifically, it asks two questions: (1) What behavioral and interbrain dynamics predict successful learning (analysis 1, see “Methods”)? (2) Can an embodied neuroscience approach be used to model the teacher’s brain to predict learner’s brain activity (and vice versa) (analysis 2, see “Methods”)? To address the first question, we used trial-level analyses to examine how learning outcomes are predicted by interpersonal neural synchrony and behavioral coordination. Specifically, we extracted wavelet coherence values from regions of interest and combined these with second-by-second behavioral annotations—namely, joint attention and mutual gaze—to build predictive models of learning success across trials and participants. To address the second question, we implemented xGLM approach to test whether a participant’s brain activity could be predicted from their partner’s brain signals and the behavioral features of the interaction. By modeling each individual’s activation as a function of their partner’s gaze, speech, head movement, physiology, and neural data, we explored whether mutual prediction occurs at the neural level and how this might differ between teaching and learning roles.

Together, these complementary approaches allow us to investigate how learning emerges from coordinated neural and behavioral activity and how interactive brains influence each other during the exchange of knowledge. By modeling both brain synchrony and individual brain responses as a function of both self and partner’s brain, behavior, and physiology, this work represents a novel and significant step forward in the neuroscience of social learning.

## Methods

### Participants

Participants were healthy young adults from the same household (data collected during the Covid-19 pandemic). Data were collected from 30 dyads. Three dyads were excluded for poor fNIRS data quality likely caused by poor scalp adherence and thick hair (Pinti et al. 2019). The final sample included 27 dyads (*n* = 54, 34 females, 19 males, 1 nonbinary, age range = 19 to 37, age mean (SD) = 26.61 (4.76), years of education mean (SD) = 19.66 (2.99)). All participants gave written consent to participate in the study and were reminded of their right to withdraw at any point. The study was approved by the University College London (UCL) Research Ethics Committee (project ID: ICN-AH-PWB-3-3-2016c).

### Material and procedure

A schematic of experimental procedure is illustrated in Fig.  1A. This study was composed of three main parts: (1) the pre-learning phase, (2) the experimental-session phase, and (3) the learning-test phase. Participants were instructed to learn facts about obscure items in their own time before the experimental session (pre-learning phase). They then taught those facts to their partner (experimental-session phase). During the experimental-session phase, half of the trials included a separator that obstructed the view of the two participants (part-view condition vs full-view condition, Fig.  1A). Both participants within each dyad were tested on their learning immediately after the experimental-session phase and a week later (learning-test phase).

Learning material was the same as De Felice et al. (2021). Sixteen items formed two learning sets (set “A” and set “B,” 8 items in each set, 2 items from each of the four categories “animals,” “antiques,” “exotic food,” and “musical instruments”). In each dyad, participants were randomly assigned to set “A” or “B.” When a participant was assigned to set “A,” they learned facts from set “A” at home. They then taught those facts to their partner during the experimental-session phase in the laboratory, and in turn, they learned facts from set “B.” To ensure the learning material was truly novel to participants, a few days before the experimental session the researcher scheduled a short call and asked each participant if they had heard of [*item*] for each of the 16 items. If any of the items were known to just one participant within the dyad, then the learning set containing the known item was assigned to that participant (ie this ensured that any pre-knowledge would contribute to the teacher performance, but *not* the learner performance). If both participants within the dyad knew about the item, then that item was retained during the experimental-session phase to ensure equal experimental length, but excluded post hoc from the analysis. At the end of the screening call and at least 48 h before the experimental-session day, participants were each sent a unique link via email, containing their learning set (pre-learning phase).

The *pre-learning phase* was conducted online (in Gorilla Experiment Builder) and lasted on average 33 min (SD = 2.7). This included a mixture of text and pictures describing 8 items. MCQs with feedback were included to facilitate memorization of the facts. Participants were instructed to complete the pre-learning phase in their own time and away from their partner, and were informed that they could repeat this phase as many times as they wanted. However, to participate to the experimental session, they had to achieve a 100% learning score.

The *experimental-session phase* was conducted in the laboratory and lasted ~1.5 h, including preparation time and debriefing. Participants sat at a circular table at ~90° angle to each other (Fig.  1B and C), and each had a box with models of the 8 items to-teach within reach. A monitor on the wall opposite the table displayed instructions for each trial and played a sound to indicate the start/end of each trial. On each trial, one participant was the “teacher” who held the model of the focal item for that trial on the table and described it for 90 s (a sound signal alerted participants about the end of the trial. If they were still speaking at that point, they were permitted to complete their thought). They were told to teach and learn as well as possible to prepare for a test after the session (explicit learning), and could choose how to use the time in each trial autonomously and freely (eg ask questions, repeat information, interrupt their partner, play with the model etc). Apart from the item model, no prompts nor scripts were given to participants during this phase.

**Fig. 2:**
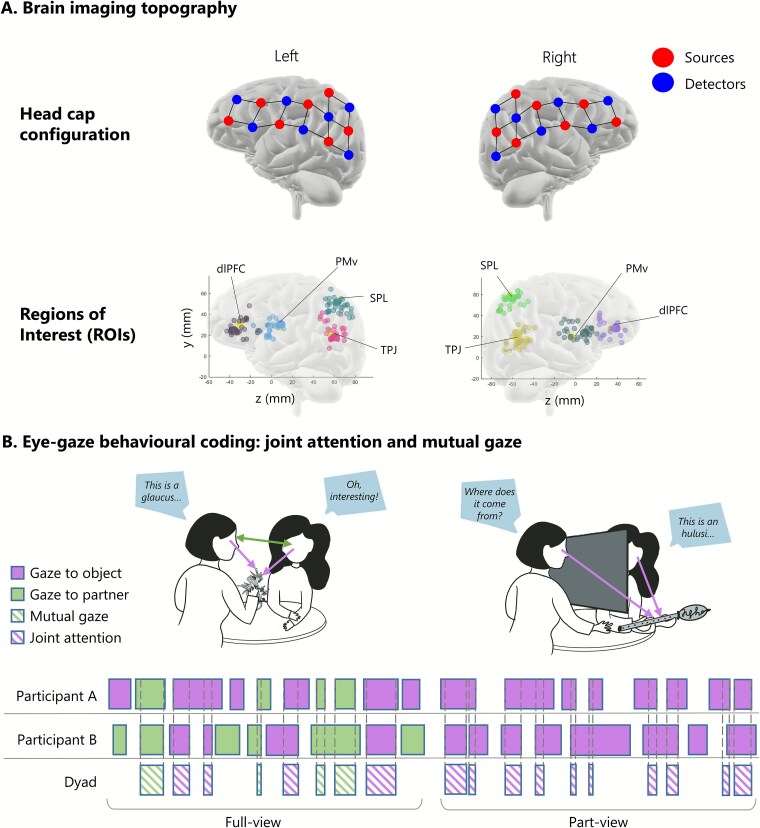
Signal processing and data analysis. A. Brain imaging topography (top) NIRS headset configuration. Optodes are divided by 7 sources and 7 detectors per hemisphere, spreading from parietal to frontal regions. This configuration forms 19 channels per hemisphere, for a total of 38 channels per participant. (bottom) Channels plotted after being assigned to one of the 8 ROIs. Each cluster represents one ROI, including dlPF: dorsolateral prefrontal cortex; PMv: ventral premotor cortex; SPL: superior parietal lobule; TPJ: temporoparietal junction. See De Felice et al. (2024) for details on how we got from optode configuration to ROIs. B. Eye-gaze behavioral coding: Joint attention and mutual gaze were coded as episodes when participants looked at each other. Joint attention was coded as episodes when both participants looked at the item at the same time. Experimental conditions include a full-view (separator “off”) and a part-view (separator “on”) condition. The full-view condition resembles a typical naturalistic interaction where both mutual gaze and joint attention episodes occur. The part-view condition creates a disrupted view where partners cannot see each other’s faces: Here, mutual gaze is not possible and only joint attention episode can occur.

Items were presented in a pseudorandom order, alternating from the set “A” and the set “B,” so that each participant was never playing the same role (student/teacher) twice consecutively. Of the 16 trials, 2 blocks of 4 trials were completed with a physical separator placed between the participants so they could not see each other’s faces but could see the table and the model item (part-view condition), and 2 blocks of 4 trials were completed with no separator between the participants (full-view condition); block order was counterbalanced (Fig.  1A and Fig.  2B).

In the *learning-test phase*, learning was measured immediately after the experimental-session phase via a free-recall questionnaire (“*can you write down all you can remember about e.g. Axolotl*?”). Once finished, participants were reimbursed for their times and reminded to complete a multiple-choice quiz by the end of the day and in a week’s time. The multiple-choice quiz link was sent to participants at the end of the experimental session and again 7 days after. This was run in Gorilla Experiment Builder and was the same as used in De Felice et al. (2021).

### Data stream acquisition

See Table S1 (Supplementary materials) for a summary of all types of data stream acquired in this study.

#### fNIRS data

Hemodynamic signals were acquired using a 56-optode (28 sources and 28 detectors, split between two heads) continuous-wave NIRS system (LABNIRS; Shimadzu Corp., Kyoto, Japan) emitting near-infrared light at three wavelengths (780, 805, and 830 nm). Each participant in a dyad had the same distribution of 38 channels over both hemispheres (7 source and 7 detectors per hemisphere, Fig.  2A), with a source–detector distance of 3 cm. Data were collected at a sampling frequency of 8.33 Hz. The location of each channel on the head as well as of five anatomical landmarks (nasion, inion, left auricular, right auricular, vertex) were recorded with a two-sensor 3D digitizer (Liberty; Polhemus, USA) and converted to MNI coordinates using NIRS-SPM (Ye et al. 2009).

#### Video recordings

Videos were recorded from three cameras connected to a single hard drive (Swann security systems). Cameras 1 and 3 were placed to capture participant A and participant B, each with a frontal view, while camera 2 was placed higher up in the middle to capture the full interaction (Fig.  1B).

#### Audio recordings

Audio was recorded at the standard frequency of 40 kHz with two lapel microphones attached to the chest of each participant.

#### Breath belts

Breathing signal was recorded at 4.4 kHz. Each participant was fitted with two breath belts (MLT1132; ADInstruments, Castle Hill, Australia) with one positioned at the abdomen and the other at the chest level to account for individual differences in breathing patterns. The stronger signal for each participant was used to extract a breathing measure.

#### Head-tracking data for nodding analysis

Head movement was tracked at 120 Hz using a Polhemus Electromagnetic Tracking system (https://polhemus.com), with a sensor placed on the forehead of each participant.

#### Multimodal data temporal alignment

All data streams were recorded continuously throughout the experimental-session phase. Data streams were recorded through different devices (see Table S1): head-tracking data were recorded through a Polhemus system in MATLAB software on a laptop; audio recordings and breath-belt data were acquired using an ADInstruments IO data acquisition box and recorded through LabChart software and brain data were recorded via the Shimadzu LABNIRS system. MATLAB and Psychtoolbox software were used to record task events (timings of trials) and send trigger signals to a NI-DAQ IO box. This, in turn, sent the trigger signals to the LabNIRS and ADInstruments box, ensuring temporal alignment across these different data streams (Fig.  1C). Video recordings were manually aligned post hoc.

### Signal processing and outcome measures

#### Brain data (fNIRS data)

Raw data were converted into .nirs using a custom-written MATLAB script. Each converted raw file was then split into two, based on the channel configuration of each individual participant within one dyad. Individual channel inspection for inclusion was assessed following the pipeline in Pinti et al. (2019). Specifically, channels were marked as “high-quality” (and included) if the heart rate oscillation was visible in the frequency spectrogram, or if light saturation artifacts and/or large motion errors were not present. Channels not passing these quality checks were excluded from further analyses.

After the data quality check and exclusion, on average each channel had 44 data points (out of 54 participants, min = 31; max = 53). For the included high-quality channels, raw intensity signals at the 3 wavelengths were preprocessed using the Homer2 toolbox. In particular, intensity data were converted into changes in optical density (function: hmrIntensity2OD). Optical densities were then corrected for motion artifacts using the wavelet-based method (function: hmrMotionCorrectWavelet, iqr = 1.5) and bandpass filtered in the range [0.01 0.4] Hz (fifth-order Butterworth filter, function: hmrBandPassFilt). Changes in HbO_2_ and HbR were calculated using the modified Beer–Lambert law assuming a fixed DPF of [666] (function: hmrOD2Conc). HbO_2_ and HbR were then combined into the “activation signal” through the CBSI approach (Cui et al. 2011; Burgess et al. 2022; Hakim et al. 2023).

Large variability in channel location across participants is common in studies using fNIRS (Zimeo Morais et al. 2018). To address this, we selected 8 ROIs (4 per hemisphere) based on relevant research literature using neurosynth database (https://neurosynth.org/). Selected ROI included dorsolateral prefrontal cortex (dlPFC), superior parietal lobe cortex (SPL), temporoparietal junction (TPJ), and ventral premotor cortex (PMv). To account for the variability in channel location across individuals and to ensure that the signal reflects the hemodynamic activity rather than noise, for each participant and ROI, the one high-quality channel closest to the center of that ROI was taken as the data for that channel and other channels were discarded. Using one channel per ROI (instead of averaging over multiple close-by channels) ensures that the fine temporal structure of the data from each channel is preserved and that anatomically homogeneous channels are pooled together for the group-level statistics. This is particularly important for cross-brain analysis. The final fNIRS analysis dataset comprised 8 ROIs per participant (see De Felice et al. 2024 for details).

#### Eye-gaze behavior (video recordings)

Video recordings of each session were coded to obtain measures of joint attention and mutual gaze. Video coding was conducted by three trained coders, who divided the dataset among them. To ensure consistency, a second coder (the first author) checked ~30% of each coder’s annotations. When discrepancies were identified, coders agreed on a shared approach and re-coded their segments. A further 30% of the revised data were then randomly re-checked. Only two rounds of checks were required for any coder. This procedure ensured inter-rater reliability and consistency across coders. Trained coders annotated the videos for each individual participant in ELAN (Wittenburg et al. 2006) and coded each time point as “gaze to object” or “gaze to partner” or neither. From these, *joint attention* was computed as the time when both participants looked at the learning object simultaneously; *mutual gaze* was computed as the time when participants looked at each other (only available for full-view trials). In addition, we computed an extra measure for *sustained attention* separately for participant A and B as the time each one of them looked at the learning object (independently on their partner’s behavior). This was to control for time participants attended to the object which was not related to the interaction (Yu and Smith 2011, 2013). See Fig.  2B for a graphical representation of the gaze coding.

#### Speech (audio recordings)

The speech wave for each participant was extracted using the “separateSpeakers” function available as a standard tool in MATLAB which separates a mixed audio track into two distinct tracks, one for each person. Each person’s audio (at 40 kHz) was rectified and smoothed with a moving average (4,000-time-point window) to obtain the audio envelope. The envelope was thresholded to classify each timepoint as “speaking” or “not speaking.” The threshold was decided ad hoc for each participant to capture the major changes in the signal. The signal was then downsampled to 1000 Hz (Matlab “downsample” function) and then downsampled again by averaging the signal in a 0.14-s window centered on each fNIRS datapoint (at 8.33 Hz). This allowed us to obtain an audio envelope on the same timescale as the fNIRS data. Implementing a gradual downsampling approach allowed us to run some regular visual checks on the data and ensured the signal remained indicative of task behavior.

#### Breathing (abdominal tracking)

Breathing data from the belt with the strongest signal were preprocessed, and breathing rate was extracted using the NeuroKit2 toolbox (Makowski et al. 2021). The preprocessing went as follows: the data were filtered using a second-order bandpass Butterworth filter (0.1 to 0.35 Hz) followed by a constant detrending. The cleaned signal was used to identify peaks and process exhalation process (peaks) and inhalation onset (troughs), which were then used to compute the respiratory rate (Khodadad et al. 2018). This was again resampled to 8.33 Hz to match the fNIRS data.

#### Nodding (head tracking)

A nod (ie a brief, typically downward movement of the head) is often used as a nonverbal signal during communication. To identify when participants performed nods, we analyzed head-movement data using the same method described by Hale and colleagues (Hale et al. 2020). Specifically, we applied the fast nod detector algorithm, which defined fast nods as pitch-axis head movements with a dominant frequency falling within the range of 1.5 to 8 Hz. Each time point in the fNIRS data was classified as “nodding” or “not nodding” based on the output of the detector.

#### Learning

Learning on each item was assessed by combining scores on the free-recall task, the immediate multiple-choice questionnaire, and the delayed multiple-choice questionnaire, each scored as percent correct over the number of facts reported by the teacher. Full details of the scoring rules are given in the Supplementary information. To ensure that performance reflected learning during the task, participants were first asked whether they already knew each item (during a pre-experiment screening call, see “Materials and procedure”), and only items reported as unknown (pre-experiment) were included in the scoring. The final learning outcome was a single continuous measure: the mean percent of correct responses across free-recall, immediate, and delayed multiple-choice questionnaires.

## Data analysis

### Analysis 1: what predicts successful learning?

This analysis tests if INS and/or social behavior can predict on which trials a participant successfully learns new information. To quantify neural synchrony, we calculated the *coherence* between the CBSI signals of the two brains within each dyad over the 8 homologous ROIs per trial, using the MATLAB R2020b function *wcoherence.* This resulted in wavelet transform coherence values over time for each trial, which were averaged across two selected frequency bands. We focused WTC analyses on homologous channel pairs to capture symmetrical brain–behavior patterns, complementing the xGLM analyses of asymmetry (analysis 2), and thereby also reducing the multiple-comparisons burden. Future studies with larger samples may extend this approach to nonhomologous pairs.

Based on previous fNIRS hyperscanning literature and theoretical and methodological considerations, we focused our analysis on the 0.03- to 0.2-Hz range (Zhang et al. 2020). We disregarded frequency components >0.2 Hz, as these would not reflect true brain signal as measured by fNIRS (the hemodynamic response has a frequency < 0.2 Hz), but rather physiological components such as respiration (Tachtsidis and Scholkmann 2016); and frequencies <0.03 Hz as these are generally linked to very low frequency noise, such as instrumental noise or vascular endothelial regulations (Yücel et al. 2016; Pinti et al. 2019). Within this range, we selected two frequency bands of interest, namely, high (0.1 to 0.2 Hz, ie 5- to 10-s period) and low (0.03 to 0.1 Hz, ie 10- to 30-s period) to capture both the faster processes involved in our task (eg turn-taking) and slower social-cognitive processes (eg mutual understanding). This decision was informed by both a general agreement in the literature that interpersonal synchrony can be spread across multiple frequencies (Nozawa et al. 2016) and that different frequencies in neuronal rhythms reflect different cognitive processes (Ward 2003; Hasson et al. 2008; Cannon et al. 2014), and more specifically previous fNIRS studies looking at brain coherence in social interaction contexts (Cui et al. 2011; De Felice et al. 2024). We obtained two average coherence values for each individual trial (high- and low-frequency value).

To identify the subset of neural and behavioral features most predictive of learning, we employed a LASSO-regularized generalized linear model (GLM) using MATLAB’s *lassoglm* function (Statistics and Machine Learning Toolbox; MATLAB R2024a). This approach imposes an L1 penalty on the regression coefficients (absolute value) according to a regularization coefficient λ, driving the weights of some regressors to zero, essentially excluding those regressors that do not contribute to the model, thereby preventing overfitting and improving generalization. We used LASSO-GLM for variable selection because it effectively handles high-dimensional, correlated predictors and enhances generalizability by reducing overfitting, making it preferable to traditional stepwise approaches (Bouchlaghem et al. 2022).

The set of candidate predictors included coherence values for each ROI in the two frequency bands (8 ROIs × 2 frequency bands, 16 terms), joint attention (seconds, 1 term), and the view condition (full-view or part-view [0/1], 1 term). Because this analysis focuses on *symmetric* signals, speech is not included in this model. The model also included all possible interaction terms, namely, view × joint attention (1 term), view × coherence in all ROIs (16 terms), and joint attention × coherence in all ROIs (16 terms). This gave us a total of 51 predicting terms. The full set of original and interaction predictors was then submitted to a LASSO-regularized generalized linear model (Gaussian family) with 10-fold cross-validation. All predictors were automatically standardized (*z*-scored) by lassoglm prior to fitting. A fixed penalty (λ = 0.001) was chosen a priori to ensure consistency and interpretability across all models, rather than allowing cross-validation to select slightly different penalties in each model. Fixing λ facilitates a direct comparison of retained predictors across models: any differences in selected features cannot be attributed to shifts in the penalty but only to genuine differences in the underlying data. A λ = 0.001 is considered to be a mild-to-moderate penalty (Friedman et al. 2010) and higher than other studies (Wittmann et al. 2025). This ensured that our model remains parsimonious without over-shrinking meaningful regressors. Nonzero coefficient weights at this λ identified the subset of predictors retained for subsequent analysis.

The selected predictors were entered as fixed effects in a linear mixed-effects model. This model also included random intercepts for item list, dyad, teacher performance, and individual learner sustained attention, to estimate unbiased effect sizes and confidence intervals. Because this model included a dataset for both view conditions (part-view and full-view), we refer to this model as the *combined* model.

**Fig. 3:**
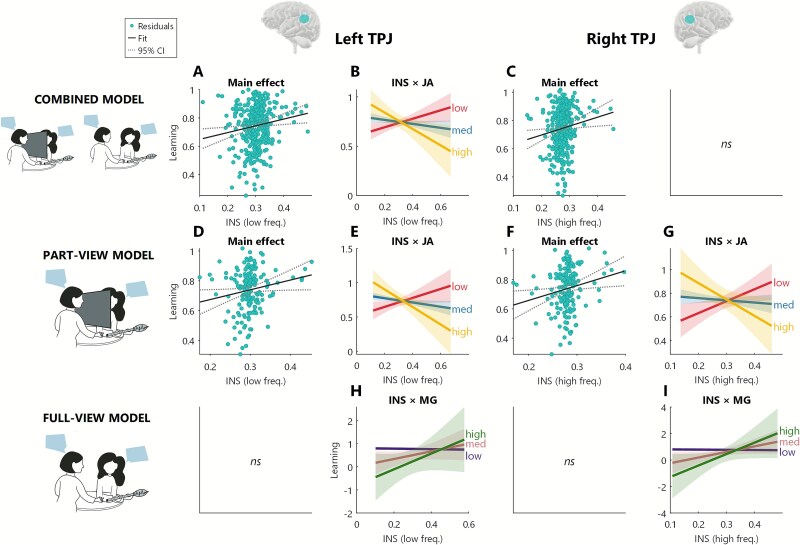
Lasso-GLM results for wavelet coherence analysis, eye-gaze behavior, and learning for TPJ. Panels A, B, D, E, H: left TPJ for main effect (A and B) and for interaction effect with joint attention (B and E) and mutual gaze (H). Panels C, F, G, I: right TPJ for main effect (C and F) and for interaction effect with joint attention (G) and mutual gaze (I).

**Fig. 4:**
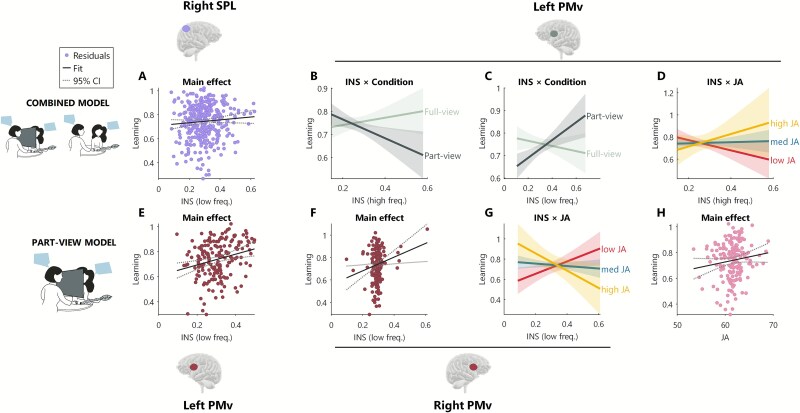
Lasso-GLM results for wavelet coherence analysis, eye-gaze behavior, and learning for bilateral PMv and right SPL. Panel A: main effect of INS in right SPL. Panels B, C, D: interaction effects for left PMv-INS with condition (B and C) and joint attention (D). Panel E: main effect of INS in left PMv. Panels F, G: main effect in right PMv (F) and interaction effect between right PMv and joint attention (G). Panel H: main effect of joint attention.

To further investigate the relationship between INS, behavior, and learning, in addition to the combined model, we repeated the above pipeline for the part-view and the full-view datasets separately, keeping the λ = 0.001. In the *full-view* model, we were able to include mutual gaze as an additional predictor; however, because this term is inherently collinear with the view condition (since mutual gaze occurred only in the full-view condition), it was omitted from the combined and part-view model. Results for these analyses are reported below and in Fig.  3 and Fig.  4.

### Analysis 2: what factors drive cross-brain effects?

To obtain a more detailed understanding of what drives the brain activity of a participant during our teaching–learning task, we used a cross-brain GLM approach. This also allows to focus on asymmetric effects that cannot be explored in the wavelet coherence models. We followed the method of Cañigueral and colleagues (Cañigueral et al. 2021) and aim to predict the brain activity of participant A from brain activity of participant B as well as from a series of other regressors accounting for behavior (nodding, speech, joint attention) and physiology (breathing) from both participant A and B. Mutual gaze was excluded from this analysis because this term is inherently collinear with the view condition. Notably, in this analysis every participant can contribute data as A and also as B, meaning that we analyze 40 distinct datasets here.

**Fig. 5:**
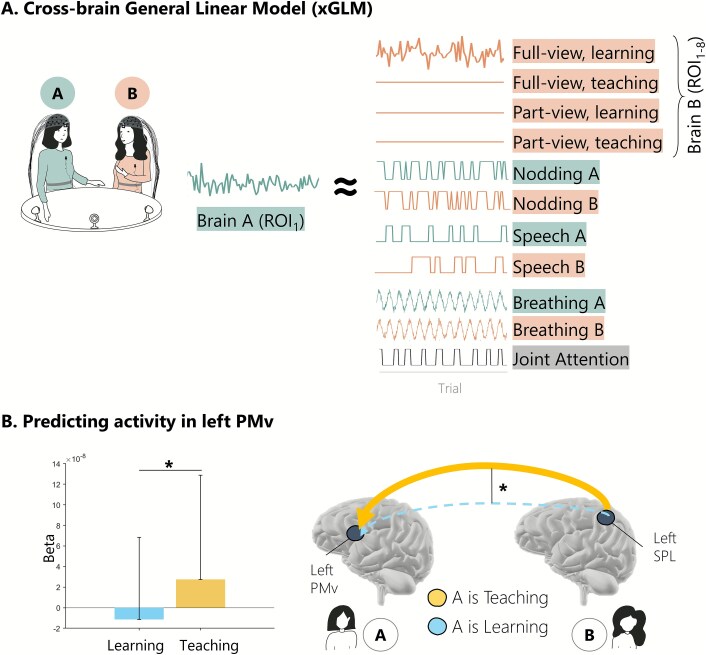
xGLM results. A. Schematic of the xGLM design matrix. A schematized version of the design matrix for one trial includes condition-specific regressors (full-view/part-view × learning/teaching), behavioral events (eg nodding, speech, breathing), and joint attention episodes. Note that for simplification, here only one trial has been illustrated, showing full-view learning condition for brain B ROI regressor containing data with the other regressors (other experimental conditions) being flat (not contributing). All data streams are time aligned within dyad members and used to predict brain activity over one ROI of the partner within each dyad. Neural time series were extracted from predefined ROIs for each participant and used to build predictive models of cross-brain effects. B. Predicting activity in left PMv. We modeled activity in participant A’s ventral premotor cortex (PMv) using regressors derived from participant B’s superior parietal lobule (SPL). Results showed a significant increase in predictive coupling from left SPL (B) to left PMv (A) during teaching (A in teacher role) compared to learning (A in learner role). Note that we modeled activity from all ROIs, but here we show only the results surviving FDR correction.

In order to capture how cross-brain coupling changes in different experimental conditions, we took each ROI regressor (eg activity of participant B in TPJ) and split it into four separate regressors reflecting activity during (1) full-view learning, (2) full-view teaching, (3) part-view learning, and (4) part-view teaching blocks. These regressors were added to the design matrix of each participant, allowing us to model person A’s brain activity in terms of their partner’s brain activity over full-/part-view and learning/teaching mode. In addition, the design matrix contained regressors for self-behavior (speaking, nodding, and breathing) and for other-behavior (speaking, nodding, breathing) as well as for joint attention. An illustration of the design matrix is in Fig.  5A.

As there are 8 ROIs in our dataset, we built 8 different design matrixes, each using one ROI from B to generate the set of “brain predictors.” We fit each of the 8 design matrices to predict the brain activity of person A and report all contrasts with FDR correction (*q* < 0.05) for multiple comparisons over the 8 ROIs. The contrasts we tested from these designs include a series of self-/other contrasts that capture asymmetry in behavior: self_speech > other_speech; self_nod > other_nod; self_breath > other breath; and also contrasts for the cross-brain effects: teach > learn and full-view > part-view.

Note that the LASSO-GLM and xGLM analyses address complementary questions: the former identifies behavioral and neural features associated with learning across dyads, while the latter estimates directed influences within individual dyads and averages these effects across the sample.

## Results

Descriptive statistics for all experimental variables are reported in Supplementary material (Table S2).

### Learning performance

Participants were able to complete the task and showed good learning performance (average score of 75.19%), with no flooring or ceiling effects. Learning performance did not differ between the part-view and the full-view condition (*t*_(53)_ = 0.75, *P* = 0.46; see Fig. S1A in Supplementary material).

### Analysis 1: what predicts successful learning?

To understand how INS and social behaviors predict learning, we report the wavelet coherence analysis combined with the sparse regression via LASSO-regularized GLM for the “combined model” (both experimental conditions combined), the “part-view model,” and the “full-view model.”

*The combined model* includes data from all trials but excludes mutual gaze as a factor (because it is confounded with the full-/part-view manipulation)*.* At the chosen penalty (λ = 0.001) with 10-fold cross-validation, the LASSO-GLM retained 29 of the 51 candidate predictors (see Table S3). When these 29 predictors were included as fixed effects in a linear mixed-effects model to predict learning (with random intercepts for item list, dyad, teacher report, and learner sustained attention), we found main effects for left TPJ (low frequency, Fig.  3A), right TPJ (high frequency, Fig.  3C), and right SPL (low frequency, Fig.  4A). All these regions showed a positive relationship to learning: the higher the INS over these regions, the better the learning. We also found a negative interaction effect between left TPJ (low frequency) and joint attention, where for trials with high joint attention, INS was negatively associated with learning, while for trials with low joint attention, INS was positively associated with learning (Fig.  3B). Left PMv showed an interaction effect with view condition which was negative for high-frequency band and positive for low-frequency band (Fig.  4B and C). Full statistics are reported in Table 1. To explore these results in more detail, we ran two separate models for the full-view and part-view trials.

**Table 1:** Combined model: sparse regression via LASSO-regularized GLM results.

	**Estimate**	**SE**	**tStat**	**DF**	***P*-value**	**Lower**	**Upper**
Intercept	0.57	0.08	7.07	332	0.00	0.41	0.73
**Main effects**							
*High-frequency band (0.1 to 0.2 Hz)*							
dlPFC left	0.02	0.10	0.16	332	0.87	−0.18	0.22
PMv right	−0.03	0.10	−0.26	332	0.80	−0.21	0.16
PMv left	−0.23	0.20	−1.20	332	0.23	−0.62	0.15
TPJ right	0.41	0.18	2.25	332	0.03	0.05	0.76
TPJ left	−0.09	0.19	−0.49	332	0.63	−0.46	0.28
*Low-frequency band (0.03 to 0.1 Hz)*							
dlPFC right	0.11	0.17	0.68	332	0.50	−0.22	0.45
dlPFC left	0.04	0.09	0.46	332	0.65	−0.14	0.23
PMv right	0.12	0.14	0.88	332	0.38	−0.15	0.39
PMv left	−0.14	0.09	−1.53	332	0.13	−0.32	0.04
TPJ right	−0.24	0.13	−1.78	332	0.08	−0.50	0.02
TPJ left	0.50	0.13	3.75	332	0.00	0.24	0.76
SPL right	0.16	0.07	2.47	332	0.01	0.03	0.29
**Interaction effects**							
*High-frequency band (0.1 to 0.2 Hz)*							
dlPFC right × JA	0.00	0.00	0.93	332	0.35	0.00	0.01
dlPFC right × view condition	0.13	0.18	0.71	332	0.48	−0.22	0.48
PMv left × JA	0.01	0.00	2.17	332	0.03	0.00	0.01
PMv left × view condition	−0.52	0.18	−2.87	332	0.00	−0.87	−0.16
TPJ right × JA	−0.01	0.00	−1.73	332	0.08	−0.01	0.00
TPJ left × JA	0.00	0.00	0.19	332	0.85	−0.01	0.01
TPJ left × view condition	−0.24	0.18	−1.31	332	0.19	−0.60	0.12
SPL left × JA	0.00	0.00	1.07	332	0.29	0.00	0.00
*Low-frequency band (0.03 to 0.1 Hz)*							
dlPFC right × JA	0.00	0.00	0.27	332	0.79	0.00	0.01
dlPFC left × view condition	−0.01	0.12	−0.08	332	0.94	−0.25	0.23
PMv right × JA	0.00	0.00	−0.32	332	0.75	−0.01	0.00
PMv left × view condition	0.43	0.13	3.20	332	0.00	0.16	0.69
TPJ right × JA	0.00	0.00	1.29	332	0.20	0.00	0.01
TPJ left × JA	−0.01	0.00	−2.92	332	0.00	−0.01	0.00
TPJ left × view condition	−0.12	0.13	−0.92	332	0.36	−0.38	0.14
SPL left × JA	0.00	0.00	−0.98	332	0.33	−0.01	0.00
SPL left × view condition	0.22	0.13	1.64	332	0.10	−0.04	0.48

*The full-view model* allows us to test if the above effects apply when participants can fully see each other and adds a predictor for mutual gaze*.* At the chosen penalty (λ = 0.001) with 10-fold cross-validation, the LASSO-GLM retained 31 of the 51 candidate predictors (see Table S4). When these 31 predictors were included as fixed effects in a linear mixed-effects model (with random intercepts for item list, dyad, teacher report, and learner sustained attention), we found an interaction effect between left TPJ (low frequency) and mutual gaze (Fig.  3H), and between right TPJ (high frequency) and MG (Fig.  3I). Both these interactions were positive: when participants exhibited higher mutual gaze on a given trial, INS over these regions was associated with higher learning. Full statistics is reported in Table 2.

**Table 2:** Full-view model: sparse regression via LASSO-regularized GLM results.

	**Estimate**	**SE**	**tStat**	**DF**	***P*-value**	**Lower**	**Upper**
**Original predictors**							
*High-frequency band (0.1 to 0.2 Hz)*							
dlPFC right	−0.04	0.14	−0.27	151	0.78	−0.32	0.24
PMv left	−0.07	0.41	−0.18	151	0.86	−0.88	0.73
TPJ right	0.23	0.32	0.73	151	0.47	−0.40	0.86
TPJ left	0.17	0.20	0.86	151	0.39	−0.22	0.57
SPL right	−0.06	0.15	−0.42	151	0.67	−0.36	0.23
SPL left	0.23	0.15	1.55	151	0.12	−0.06	0.53
*Low-frequency band (0.03 to 0.1 Hz)*							
dlPFC right	−0.04	0.27	−0.16	151	0.88	−0.57	0.49
dlPFC left	0.12	0.21	0.60	151	0.55	−0.29	0.53
PMv right	0.02	0.10	0.16	151	0.87	−0.18	0.21
PMv left	−0.04	0.25	−0.15	151	0.88	−0.52	0.45
TPJ right	−0.27	0.23	−1.16	151	0.25	−0.72	0.19
SPL right	0.12	0.15	0.75	151	0.46	−0.19	0.42
SPL left	−0.11	0.12	−0.90	151	0.37	−0.34	0.13
*Behavior*							
MG	−0.02	0.01	−1.47	151	0.14	−0.04	0.01
**Interaction predictors**							
*High-frequency band (0.1 to 0.2 Hz)*							
dlPFC left × JA	0.00	0.00	1.02	151	0.31	0.00	0.01
dlPFC left × MG	−0.03	0.02	−1.36	151	0.18	−0.07	0.01
PMv right × MG	0.01	0.01	1.02	151	0.31	−0.01	0.04
PMv left × JA	0.00	0.01	0.68	151	0.50	−0.01	0.02
PMv left × MG	0.00	0.02	−0.02	151	0.98	−0.04	0.04
TPJ right × JA	−0.01	0.01	−1.45	151	0.15	−0.02	0.00
TPJ right × MG	0.06	0.02	2.43	151	0.02	0.01	0.10
TPJ left × MG	−0.02	0.02	−1.19	151	0.23	−0.06	0.02
*Low-frequency band (0.03 to 0.1 Hz)*							
dlPFC right × JA	0.00	0.01	0.63	151	0.53	−0.01	0.01
dlPFC left × JA	0.00	0.00	−0.43	151	0.67	−0.01	0.01
PMv left × JA	0.00	0.00	−0.46	151	0.65	−0.01	0.01
PMv left × MG	0.00	0.01	−0.11	151	0.91	−0.03	0.03
TPJ right × JA	0.00	0.00	0.62	151	0.54	−0.01	0.01
TPJ right × MG	0.00	0.01	−0.12	151	0.90	−0.03	0.03
TPJ left × JA	0.00	0.00	−0.93	151	0.35	−0.01	0.00
TPJ left × MG	0.03	0.01	2.19	151	0.03	0.00	0.05
SPL right × MG	0.01	0.01	0.75	151	0.46	−0.02	0.03

*The part-view model* includes only the part-view trials and omits the mutual gaze predictor*.* At the chosen penalty (λ = 0.001) with 10-fold cross-validation, the LASSO-GLM retained 24 of the 33 candidate predictors (see Table S5). When these 24 predictors were included as fixed effects in a linear mixed-effects model to predict learning (with random intercepts for item list, dyad, teacher report, and learner sustained attention), we found a main positive effect of joint attention (Fig.  4G). In addition, we found a main effect of left TPJ (low frequency, Fig.  3D), right TPJ (high frequency, Fig.  3F), and left and right PMv (low frequency, Fig.  4D and E). All these regions showed a positive relationship to learning: the higher the INS over these regions, the better the learning. We also found a negative interaction effect between left TPJ (low frequency) and joint attention (Fig.  3E), right TPJ (high frequency) and joint attention (Fig.  3G), and right PMv (low frequency) and joint attention (Fig.  4F), where for trials with high joint attention, INS over these regions was negatively associated with learning, while for trials with low joint attention, INS over these regions was positively associated with learning. Full statistics are reported in Table 3.

**Table 3:** Part-view model: sparse regression via LASSO-regularized GLM results.

	**Estimate**	**SE**	**tStat**	**DF**	***P*-value**	**Lower**	**Upper**
**Original predictors**							
*High-frequency band (0.1 to 0.2 Hz)*							
dlPFC left	0.07	0.16	0.42	154	0.68	−0.25	0.38
PMv right	0.18	0.32	0.57	154	0.57	−0.45	0.81
PMv left	−0.45	0.34	−1.31	154	0.19	−1.13	0.23
TPJ right	0.88	0.33	2.66	154	0.01	0.23	1.54
TPJ left	−0.14	0.36	−0.40	154	0.69	−0.85	0.57
SPL right	−0.26	0.14	−1.84	154	0.07	−0.54	0.02
SPL left	−0.33	0.41	−0.81	154	0.42	−1.15	0.48
*Low-frequency band (0.03 to 0.1 Hz)*							
dlPFC right							
PMv right	0.23	0.28	0.82	154	0.42	−0.32	0.78
PMv left	0.51	0.22	2.32	154	0.02	0.08	0.95
TPJ left	0.32	0.11	2.90	154	0.00	0.10	0.54
SPL left	0.73	0.27	2.77	154	0.01	0.21	1.26
*Behavior*							
JA	0.01	0.00	2.37	154	0.02	0.00	0.02
**Interaction predictors**							
*High-frequency band (0.1 to 0.2 Hz)*							
PMv right × JA	0.00	0.00	−0.84	154	0.40	−0.01	0.01
PMv left × JA	0.00	0.00	0.41	154	0.68	−0.01	0.01
TPJ right × JA	−0.01	0.00	−2.60	154	0.01	−0.02	0.00
TPJ left × JA	0.00	0.01	−0.52	154	0.60	−0.01	0.01
SPL left × JA	0.01	0.01	0.85	154	0.40	−0.01	0.02
*Low-frequency band (0.03 to 0.1 Hz)*							
dlPFC right × JA	0.00	0.00	−0.01	154	0.99	−0.01	0.01
dlPFC left × JA	0.00	0.00	0.10	154	0.92	0.00	0.00
PMv right × JA	−0.01	0.00	−2.14	154	0.03	−0.01	0.00
TPJ right × JA	0.00	0.00	−0.94	154	0.35	0.00	0.00
TPJ left × JA	−0.01	0.00	−3.23	154	0.00	−0.02	0.00
SPL right × JA	0.00	0.00	1.37	154	0.17	0.00	0.00

In summary, these three models show that social behaviors and INS measures (particularly in TPJ and PMv) can predict learning, but that INS interacts with gaze behaviors and the viewing conditions in a complex way. First, we found a consistent pattern over bilateral TPJ and PMv by which INS negatively interacts with joint attention to predict learning: for moments of high joint attention, INS was negatively associated with learning, while the opposite was true during moments of low joint attention, when high INS was associated with high learning. In contrast, mutual gaze showed a positive interaction with INS: high INS during moments of high mutual gaze was associated with high learning. In addition, we observed ambiguous patterns in the relationship between INS in the left PMv and learning, as the direction of the association reversed across conditions and varied further across frequency bands. We asked whether this could be related to changes in behavior between the different conditions. To explore this possibility, we calculated the speech duration and turn-taking of teacher and learner separately across conditions: we found that in the part-view condition (compared to full-view), teacher showed a trend (approaching significance) of speaking more, possibly to compensate for the lack of visual cues in this condition (Supplementary material  Fig. S1B). Speech is one of the important asymmetric behaviors in this task, but the wavelet coherence approach is not able to capture asymmetric effects that differ within a dyad. Instead, symmetric effects could be captured by the xGLM analysis described below.

### Analysis 2: what factors drive cross-brain effects?

When predicting the brain activity of participant A based on both brain and behavior during the task, we found a robust effect of self-speech in the left SPL. That is, brain activity was greater for self-speech than other-speech in the left SPL, and this effect was found for several different ROI models (specifically when the model was built with ROI regressor being either right PMv, left PMv, right TPJ, right SPL, or left SPL). The consistency of this effect despite the other regressors in the model suggests a robust relationship between left SPL and self-speech. There were no effects of self-nodding or self-breathing.

In the examination of the cross-brain effects, we found a relationship between left SPL and left PMv across the two people within the dyad which was modulated by the participant’s role in the task. Specifically, left SPL in participant B could predict activity of left PMv in participant A significantly better when A was teaching (compared to when A was learning; *t*_(2,22)_ = 3.18, *P* = 0.03 FDR corrected, Fig.  4B). In other words, the learner’s brain was attuned to the teacher’s brain, and specifically to the teacher’s left PMv. Full statistics for all models are reported in Tables S5–S12.

## Discussion

The aim of this study was to identify markers of successful interactive learning. To do so, we adopted a highly ecological design (Vigliocco et al. 2024) and a multimodal approach. Using fNIRS hyperscanning, we measured brain activity from 27 learner–teacher pairs simultaneously as they exchanged factual information about unfamiliar objects (eg exotic animals). At the same time, we measured teacher’s and learner’s speech, breathing, and head nodding (see Fig.  1B) and coded their eye-gaze behavior (see Fig.  2B). We analyzed these data in two ways: first, we used wavelet coherence analysis to compute INS over dlPFC, PMv, TPJ, and SPL bilaterally, as well as measuring the time each pair spent in joint attention and mutual gaze. This analysis revealed a main effect of both INS and joint attention in predicting successful learning. Furthermore, we found complex and distinctive interactions between eye-gaze behaviors and INS: joint attention was associated with better learning when bilateral TPJ-INS and right PMv-INS was low and left PMv-INS was high, whereas mutual gaze was associated with better learning when TPJ-INS was high. Second, we integrated our complex multimodal dataset into a xGLM (Hamilton 2021) to explore the cognitive mechanisms that underlie the teaching–learning interaction. This allows us to examine asymmetric effects of speaking/listening and teaching/learning. We found strong effects of speaking: participants showed greater activity in left SPL during self-speaking compared to listening. We also found that left PMv activity in one participant (A) was predicted by left SPL activity in their partner (B) specifically when A was teaching. We discuss the results from each of these analyses separately and in details below.

### TPJ and gaze behavior together predict learning

Our main finding from the wavelet coherence analysis points to learner–teacher INS over bilateral TPJ as a marker of learning (see Fig.  3). For both the combined model and the part-view model, we found that more INS over left and right TPJ was associated with more learning. These findings align with previous results. TPJ represents a key hub within the social brain network, supporting theory of mind and shared understanding (Doricchi et al. 2022; Jiang et al. 2015; Zheng et al. 2018), as well as being important in predicting other’s action (Kayhan et al. 2022) and monitoring verbal exchange and turn-taking in real-time communication (Cañigueral et al. 2021; Jiang et al. 2012; Liu et al. 2019). More relevant for the present study, TPJ has been reported to be among the core areas to show brain synchrony during learner–teacher interaction in a number of studies on learning (Pan et al. 2020b; Zheng et al. 2018, Zhang et al. 2020).

While our data suggest that TPJ is a core region for predicting learning, we also find similar patterns in other brain areas. A main effect of INS predicting learning was found in right PMv, left PMv, and right SPL. Right PMv plays a central role in action understanding and interpersonal coordination, as it forms part of the mirror neuron system implicated in encoding both executed and observed actions (Rizzolatti and Craighero 2004; Fiebach and Schubotz 2006), while left PMv supports language production and comprehension (Perron et al. 2024). SPL has been shown to be important to monitor social space and discriminate between self and other action (Fujii et al. 2008; Rizzolatti and Sinigaglia 2010). In the context of our study, the finding that higher INS in bilateral PMv and right SPL correlates with better learning may reflect enhanced alignment of visuospatial and sensorimotor representations between teacher and learner. During effective learning episodes, participants may not only be sharing cognitive content but also implicitly mirroring each other by encoding and aligning visuospatial information, such as gestures and gaze direction, supporting information exchange and learning.

Joint attention was also positively associated with learning in the part-view model, consistently with studies establishing joint attention as a hallmark of good conversations (Garrod and Pickering 2004; Richardson et al. 2007) and successful learning (Schertz et al. 2013; Striano et al. 2006).

However and most importantly, no previous studies have examined how brain activity and behavior *together* support learning. We report a complex pattern where, for successful learning, joint attention and mutual gaze shape INS differently over different brain regions. Trials with higher learning were associated with (1) *high* joint attention and *low* INS in bilateral TPJ and right PMv, and *high* INS in left PMV; and (2) *high* mutual gaze and *high* INS in bilateral TPJ.

These opposing interaction patterns are not easily explained by a simple trade-off between mutual gaze and joint attention. Specifically, the possibility that joint attention might have “crowded out” or replaced mutual gaze does not hold because in the part-view conditions—where the joint attention interaction was observed—participants could not see each other’s faces and therefore could not engage in mutual gaze at all. Additionally, the negative association between INS and learning during moments of high joint attention cannot be attributed to ceiling effects in the learning measure: in the full-view condition, increased mutual gaze and higher INS were still associated with *additional* improvements in learning, indicating that the measure remained sensitive to performance differences. Thus, we must explore other explanations.

We propose that these results reflect distinct *informational uptake* and *mutual grounding* dynamics, which arise spontaneously in a teacher–learner conversation. The *informational uptake* dynamic occurs when one partner (typically the teacher) introduces novel content that prompts knowledge updating in the other (typically the learner). When such moments coincide with high joint attention (ie when both partners focus on the object of learning), they may lead to enhanced learning outcomes. Because the content is new and not yet jointly represented, INS may be relatively low in regions associated with predicting others’ actions and mental states, such as the TPJ (Frith 2008; Van Overwalle 2009; Schurz et al. 2017), and in areas involved in action understanding (Rizzolatti and Craighero 2004; Fiebach and Schubotz 2006) and interpersonal communication (Fujii et al. 2008; Chang et al. 2023), such as the right PMv. At the same time, INS may be high in regions involved in speech production and comprehension, such as the left PMv (Perron et al. 2024), where teacher and learner may be jointly processing verbal information, either as speaker or listener. Notably, the joint attention × INS interaction observed over the left PMv occurs in the high-frequency band (0.1 to 0.2 Hz), corresponding to 5- to 10-s temporal windows, which aligns with phrase-level timing in natural language (Arnon and Snider 2010). Previous single-brain studies have consistently demonstrated the role of PMv in phonological and articulatory processing (Tremblay and Small 2011), including sensorimotor mapping during naturalistic speech in real-life noise environments (Glanz (Iljina et al. 2018). In line with this, evidence from dual-brain studies with fMRI show that during natural storytelling, speaker–listener coupling emerges over the left inferior frontal/premotor regions, and the extent of this coupling predicts comprehension (Stephens et al. 2010). During face-to-face interactions, fNIRS hyperscanning studies report increased INS over the left inferior frontal cortex, overlapping PMv, with coupling reduced in monologue or back-to-back controls (Jiang et al. 2012; Hirsch et al. 2018). Taken together, these findings demonstrate that PMv is a key hub to support communicative interactions. This is consistent with the interpretation that INS in this region may reflect verbal information attunement between teacher and learner as they jointly attend to the learning content, resulting in more effective learning.

In contrast, the *mutual grounding* dynamic involves the reinforcement, clarification, and possibly rehearsal of previously shared information. These moments are characterized by a shift from knowledge acquisition to shared understanding, as partners align on the meaning or relevance of what has been communicated. Previous work demonstrated that neural synchrony can index mutual grounding and consensus (Sievers et al. 2024). Because mutual grounding involves the negotiation and reinforcement of already shared content, it would be associated with higher INS, particularly in regions involved in conceptual alignment and shared understanding, such as the TPJ (Frith 2008; Van Overwalle 2009; Schurz et al. 2017). Notably, these moments also tend to co-occur with high mutual gaze, consistent with increased social engagement. Together, high INS and mutual gaze may reflect a state of interpersonal alignment, where both partners are actively co-constructing a common representational space, facilitating consolidation and retention of the learning material. Importantly, *informational uptake* and *mutual grounding* dynamics are unlikely to unfold in a fixed sequence or as discrete phases, but may alternate and reoccur throughout the interaction in a fluid, context-dependent manner. We recognize the partially speculative nature of this interpretation, which serves to situate complex multimodal data and to inform more targeted hypothesis-driven research in the future.

We note that our finding that TPJ-INS and mutual gaze act together to support learning (mutual grounding dynamic) runs counter to previous suggestions that mutual gaze desynchronizes an interaction (Wohltjen and Wheatley 2021). However, this is in line with social learning studies showing that eye contact matters for learning in infants (Yu and Smith 2011, 2013) and adults (De Felice et al. 2021). In fact, the interaction between INS and eye-gaze behavior may follow a distinctive pattern when examined within the specific context of pedagogical interactions. Unlike other forms of social exchange, pedagogical interactions are structured: interlocutors adopt clearly defined roles (teacher and learner) and share an explicit, goal-directed objective, namely, the transmission and acquisition of knowledge. This context shapes expectations and behaviors, giving rise to a complex interplay of dynamics including informational uptake and mutual grounding. As such, the interplay between INS and eye-gaze may be finely tuned to support learning, potentially differing in both form and function from interactions in less structured or noninstructional social contexts.

In addition, we observed a complex pattern in the left PMv: in the high-frequency band, higher INS was associated with greater learning in the full-view condition but with reduced learning in the part-view condition. The opposite trend emerges for the low-frequency band (see Fig.  4B and Fig.  4C). These results may reflect different interaction dynamics between teacher and learner across conditions, with high- and low-frequency bands possibly being markers of different cognitive processes (Hasson et al. 2008). The left PMv is a region highly involved in speech perception and production (Perron et al. 2024): teacher and learner may have implicitly changed their verbal behavior based on whether they could fully see their interlocutor or not. To explore this possibility, we calculated the speech duration and turn-taking of teacher and learner separately across conditions: we found that in the part-view condition (compared to full-view), teacher showed a trend (approaching significance) of speaking more, possibly to compensate for the lack of visual cues in this condition (Supplementary material  Fig. S1B).

### Effects of speech and teacher/learner role in the left hemisphere

The INS analysis described above offers a powerful tool to identify which factors predict learning after a social interaction has taken place. However, this approach is best suited for examining *symmetric* behaviors—those that occur simultaneously and similarly in both participants, such as mutual gaze or joint attention. While these behaviors are central to social engagement, many key elements of naturalistic interaction, particularly in pedagogical settings, are inherently *asymmetric*. For example, speaking and listening rarely occur simultaneously, and the roles of teacher and learner are structurally distinct by design. These asymmetries pose a challenge for current models of INS, which typically consider reciprocal and temporally aligned signals.

To address this, we conducted a second analysis using a xGLM. This allowed us to explore how asymmetric behaviors shape interbrain dynamics during our learning task. This analysis revealed strong effects of speaking: participants showed greater activity in the left SPL during self-speaking compared to listening. This aligns with prior hyperscanning evidence linking SPL to speech production within the left-hemisphere language network during face-to-face conversation (Hirsch et al. 2018, 2021), possibly reflecting self-monitoring processes. Interestingly, we did not observe a similar effect in the left ventral premotor cortex (PMv), which might have been expected given its role in speech. One possible explanation is that PMv is active during both speaking and listening, perhaps reflecting predictive processing of conversational patterns, resulting in comparable activation across conditions.

More interestingly, we found a cross-brain effect linking SPL and PMv that depended on participants’ roles. Specifically, left PMv activity in one participant (A) was predicted by left SPL activity in their partner (B) *only* when A was teaching. This effect disappeared when A was learning. These findings suggest a coordinated, role-dependent coupling within the left-lateralized language system, where SPL and PMv may work together to support effective communication from teacher to learner. Importantly, while the xGLM identifies statistical dependencies between brains, it does not imply causal directionality. Although we observed a statistically predictive relationship from the learner’s left SPL to the teacher’s left PMv, it remains plausible that the functional influence flows in the opposite direction, ie originating in the teacher’s PMv (possibly as they speak) and guiding predictive processing in the learner’s SPL. Methods with a higher temporal resolution like EEG or ECoG should further test this possibility (Goldstein et al. 2024; Zada et al. 2024).

Finally, the xGLM included additional regressors for nodding, breathing, and gaze behavior. None of these showed significant effects, suggesting that our results are not confounded by low-level physiological or motion-related artifacts. This directly addresses one of the most common and serious criticisms of hyperscanning studies, namely, that observed INS may simply reflect shared sensory input, coordinated motor activity, and/or other external factors rather than true neural coupling (Holroyd 2022). By statistically modeling and controlling for a broad set of behavioral covariates, we demonstrate that the observed cross-brain effects are not reducible to these simpler explanations. Instead, they likely reflect higher-order cognitive coordination and mutual prediction between individuals engaged in real-time, goal-directed interaction. In doing so, our approach responds to key methodological concerns in the hyperscanning literature and highlights the value of using fine-grained behavioral regressors in combination with cross-brain modeling to disambiguate the sources of INS (Hamilton 2021; De Felice et al. 2025).

### Strengths and limitations

This study offers a rare example of how brain activity, behavior, and physiology can be jointly modeled during real-world social interaction. By combining fNIRS hyperscanning with fine-grained behavioral and physiological measures in a naturalistic social learning task, we provide novel insights into the neurocognitive mechanisms that underpin social learning. A major strength of our approach lies in its ecological and multimodal design, which allowed us to examine learning as a product of reciprocal brain–body dynamics, rather than isolating neural activity from the social context in which it unfolds.

Specifically, we show that INS, particularly over bilateral TPJ and PMv, is positively associated with learning success, and that these neural markers interact in distinct ways with key social behaviors such as joint attention and mutual gaze. Furthermore, our use of a xGLM framework demonstrates that neural activity in one participant can be predicted by their partner’s brain and behavior, highlighting the relational and embodied nature of real-time teaching and learning. This addresses a persistent challenge in the hyperscanning literature: distinguishing genuine neural coupling from co-occurring but independent sensorimotor signals. In fact, the recent shift toward second-person and relational neuroscience focus (Redcay and Schilbach 2019; De Felice et al. 2025; Schilbach and Redcay 2025) has often been anchored in a brain-centric framework, overlooking behavioral signals. As a result, prior studies have struggled to disentangle whether neural coupling reflects genuine social-cognitive alignment or merely co-occurring sensorimotor inputs (Hamilton 2021; Novembre and Iannetti 2021; Holroyd 2022). By capturing real-time behavioral exchanges alongside neural activity, our study offers a more complete account of the mechanisms that support successful social learning.

Taken together, our complementary analytic approach, linking neural synchrony to learning outcomes and identifying asymmetric interbrain dependencies, provides a richer account of the mechanisms that support social learning. This work represents a step forward for the field of social neuroscience, offering both theoretical advancement and a methodological blueprint for studying the interactive brain as it operates in naturalistic contexts (Vigliocco et al. 2024).

However, several limitations should be acknowledged. First, the exploratory nature of this work, while appropriate given the novelty of the dataset and methods, makes it difficult to apply strong a priori hypotheses or clear causal interpretations. Our analytic strategy, particularly the use of LASSO-GLM, is designed to prevent overfitting, but future studies with larger samples will be needed to validate the specific regional effects and interaction patterns we report. Second, our sample size is modest compared with some recent studies (Li et al. 2025), but broadly comparable to many previous hyperscanning studies on social interaction (Hakim et al. 2023; De Felice et al. 2025). Each dyad contributed multiple interactional episodes (16 trials), leading to a rich dataset with substantial within- and between-dyad variance. To reduce the risk of overfitting and feature-selection instability, we combined LASSO regularization with cross-validation and subsequent mixed-effects modeling. Nonetheless, future work with larger samples will be important to further test the generalizability of these findings. Third, although fNIRS offers good spatial resolution for cortical surface activity, it is inherently limited in its depth penetration and may miss important contributions from deeper or subcortical structures. In addition, while this study of dyadic interaction provides a good framework to build model of naturalistic conversation, the study of interactive learning should be extended to group dynamics, in order to make conclusions that can apply to educational setting more similar to the real-world (eg classroom). Finally, all dyads were composed of household members due to the Covid-19 pandemic constraints (when data were collected). Relational duration and closeness may have influenced INS (Parkinson et al. 2018; De Felice et al. 2025). Future studies should test whether these effects generalize to interactions between unfamiliar partners and explore how social relationships shape the brain–behavior coupling that supports learning.

Future work should aim to replicate and extend these findings in larger and more diverse samples. In particular, task manipulations that directly vary social dynamics—such as turn-taking, gesture use, or the availability of visual cues—could help to tease apart causal relationships between behavior, INS, and learning. In addition, it would be particularly interesting to study these questions in adolescence, a sensitive period for brain development, particularly for social cognition (Blakemore 2008), and a time when relational social networks are notably important for optimal development, mental health, and wellbeing (Fuhrmann et al. 2015; Becht et al. 2021). Furthermore, the integration of other modalities (eg EEG, eye-tracking) could offer more granular insights into the temporal coordination of brain and behavior.

A key contribution of our study is to move beyond the predominant literature, in which social behavior and its relationship with neural activation have often been overlooked, and to establish social behavior as a core component of social learning models alongside neural activation. Our findings demonstrate that social behavior interacts with neural activation in ways that may be characteristic of teacher–learner exchanges to support learning. At the same time, we acknowledge that our data cannot determine the directionality of these relationships: in future studies, the xGLM framework introduced here could be extended to examine directional coupling using causality methods (eg Granger Causality, Dynamic Causal Modeling, brain stimulation; Li et al. 2025; Novembre and Iannetti 2021), potentially offering a mechanistic account of how mutual understanding and shared knowledge emerge from social interaction.

In conclusion, this study advances our understanding of the embodied neural mechanisms underlying social learning by showing that interbrain synchrony over key social-cognitive regions (TPJ, PMv) is linked to learning success, and that this synchrony interacts dynamically with key social behaviors such as joint attention, mutual gaze, and teacher/learner asymmetries. By studying naturalistic interaction with rigorous modeling of brain–behavior dynamics, integrating predictive and cross-brain analyses, this study demonstrates that learning emerges not only from individual cognition, but from the reciprocal coordination of brain and behavioral signals across people. This work paves the way for future research into how shared cognition operates in real-world educational and communicative settings.

## Supplementary Material

SupplementaryMaterial_bhaf323
