# Supplementary material for: Learning with others: teacher–learner brain synchrony depends on mutual gaze and joint attention
Source: Cereb Cortex. 2025 Dec 1;35(12):bhaf323. doi: 10.1093/cercor/bhaf323 (PMC12667270; doi:10.1093/cercor/bhaf323)
Supplement: SupplementaryMaterial_bhaf323 [file supplementarymaterial_bhaf323.docx]

#

# **SUPPLEMENTARY MATERIALS**

Learning with others: teacher–learner brain synchrony depends on mutual gaze and joint attention

De Felice, S.^1,2^**^*^**, Di Ciò, F.^2^, Tompkins, D.^2,3^, Hakim, U.^4^, Pinti, P.^5^, Vigliocco, G.^6^, Hamilton, A. F. de C.^2^

1. Department of Psychology, University of Cambridge, Cambridge, UK
2. Institute of Cognitive Neuroscience, University College London, London, UK
3. Department of Clinical, Educational and Health Psychology, University College London, UK
4. Department of Medical Physics and Biomedical Engineering, University College London, UK
5. Centre for Brain and Cognitive Development, Department of Psychological Sciences, Birbeck, University of London, UK
6. Psychology and Language Science, University College London, London, UK

**^*^**Corresponding author
Email: [sd2035@cam.ac.uk](mailto:sd2035@cam.ac.uk)
Mail address: Old Cavendish Building
Rayleigh Wing, Free School Lane
CB2 3RF Cambridge, UK

Table S1. Data stream list.

Data stream with associated device for acquisition, signal and outcome measures, and information on analysis relevance.

| **Data stream** | **Device** | **Signal** | **Outcome measure** | **Analysis 1 (wtc)** | **Analysis 2 (xGLM)** |
| --- | --- | --- | --- | --- | --- |
| fNIRS data | LabNIRS | Hemodynamic response | Brain synchrony (analysis 1) / Brain activity (analysis 2) | ✓ | ✓ |
| Video recordings | Camera (x3) | Eye-gaze behaviour | Joint Attention, Mutual-gaze | ✓ | ✓ |
| Audio recordings | Label microphones (x2) | Speech | Conversational patterns |  | ✓ |
| Abdominal tracking | Belts | Physiology | Breathing |  | ✓ |
| Head tracking | Polhemus | Head orientation and movement | Nodding |  | ✓ |

#### Scoring of performance on the learning task

Out of a total of 432 trials (whole sample), 16 trials were excluded based on participants’ pre-knowledge screening. Learning performance on the included trials was scored from MCQs (immediate and delay) and free recall questionnaire. For MCQs performance, a score of 0 (incorrect) or 1 (correct) was assigned to each question (five per item), and a global score was then computed for each item (max score 5). With regards to free recall, an MSc postgraduate was trained to score the content recalled (‘student performance’). 1 point was given for every fact correctly recalled (each item had a total of 15 facts). If facts were reported only partially, these were scored as 0.5. When facts were not recalled entirely, or something else was reported altogether, this was scored as 0.

In some instances, students misremembered the item’s name while still remembering facts about that item: e.g. reporting facts about *Anhinga* while referring to it as *Axolotl*. Awarding 0 to these cases would have unfairly assessed learning performance: the student recalled considerable information and only failed to remember the target name for those facts. To ensure a fair scoring, we added an additional scoring section, referred to as ‘Correct name-description association’. Students scored 1 on this section if they reported facts about an object and referred to it with the correct name (e.g. reporting facts about *Anhinga* and also referring to it as *Anhinga*). In cases where participants recalled information about an object but under the wrong name, they were awarded 0 for ‘Correct Name-Description Association’, but marked normally for the other fifteen facts.

In addition, the audio-recordings of all dyads were scored for how many facts were provided by the teacher in each trial (‘teacher performance’). This allowed us to account for variability in learning caused by variation in teaching quality which arose because our procedure prioritised ecological validity and encouraged free-flowing interaction between participants. Having a measure of ‘teacher performance’ alongside the ‘student performance’ allowed us to compute a rigorous measure of learning (student performance/teacher performance), which reflected what the student was truly taught during a naturalistic teacher-student interaction. In some cases, facts were recalled when these were not discussed verbally, but were observed visually e.g. the colour of a fruit. In these cases, facts scored a point for both student performance and teacher performance.

Overall, learning was characterised by a single score calculated as the average of the scores in the free recall test, the immediate multiple choice test and the delayed multiple choice test. Scores on each of these three tests were expressed as a % of the maximum available points given the constraints listed above, so that the total learning score for each person and each item gives the best estimate of how much the participant has learnt about that item.

Table S2. Descriptive statistics for all experimental variables

MCQ: multiple-choice question; dlPFC: dorsolateral pre-frontal cortex; vPMC: ventral pre-motor cortex; TPJ: temporo-parietal junction; SPL: superior parietal lobe; wtc: wavelet transform coherence; JA: joint attention.

|  | *Combined* | | *Part-view* | | | *Full-view* | | |
| --- | --- | --- | --- | --- | --- | --- | --- | --- |
| Variable | Mean | SD | | Mean | SD | | Mean | SD |
| Learning immediate free-recall (%) | .56 | .22 | | .54 | .23 | | .57 | .22 |
| Learning immediate MCQ (%) | .87 | .18 | | .88 | .17 | | .86 | .17 |
| Learning +1week MCQ (%) | .83 | .20 | | .82 | .21 | | .84 | .20 |
| Learning average (%) | .75 | .16 | | .75 | .16 | | .76 | .15 |
| MG (sec) | -- | -- | | -- | -- | |  |  |
| JA (sec) | 53.10 | 27.84 | | 61.55 | 28.88 | | 44.84 | 24.15 |
| Sustained Attention learner (sec) | 69 | 29.18 | | 76.17 | 26.34 | | 61.98 | 30.16 |
| dlPFC right high-freq (wtc) | .27 | .07 | | .27 | .07 | | .26 | .08 |
| dlPFC left high-freq (wtc) | .27 | .07 | | .26 | .06 | | .27 | .07 |
| vPMC right high-freq (wtc) | .26 | .07 | | .26 | .07 | | .26 | .07 |
| vPMC left high-freq (wtc) | .28 | .07 | | .28 | .07 | | .28 | .07 |
| TPJ right high-freq (wtc) | .28 | .07 | | .28 | .07 | | .27 | .07 |
| TPJ left high-freq (wtc) | .27 | .06 | | .26 | .06 | | .28 | .07 |
| SPL right high-freq (wtc) | .27 | .07 | | .27 | .08 | | .27 | .06 |
| SPL left high-freq (wtc) | .27 | .07 | | .27 | .07 | | .26 | .06 |
| dlPFC right low-freq (wtc) | .30 | .10 | | .30 | .10 | | .29 | .10 |
| dlPFC left low-freq (wtc) | .31 | .10 | | .32 | .11 | | .30 | .10 |
| vPMC right low-freq (wtc) | .29 | .10 | | .29 | .19 | | .29 | .09 |
| vPMC left low-freq (wtc) | .31 | .09 | | .31 | .09 | | .31 | .09 |
| TPJ right low-freq (wtc) | .30 | .10 | | .30 | .09 | | .30 | .10 |
| TPJ left low-freq (wtc) | .29 | .10 | | .29 | .10 | | .29 | .10 |
| SPL right low-freq (wtc) | .30 | .10 | | .30 | .11 | | .30 | .10 |
| SPL left low-freq (wtc) | .29 | .09 | | .29 | .10 | | .28 | .09 |

Table S3. LASSO‐GLM survived predictors for the Combined model.

The Big model was run on both part-view and full-view datasets, and included ‘view condition’ as a regressor. dlPFC: dorsolateral pre-frontal cortex; vPMC: ventral pre-motor cortex; TPJ: temporo-parietal junction; SPL: superior parietal lobe; JA: joint attention.

|  | **Predictor** | **Coefficient** | **Lambda Used** |
| --- | --- | --- | --- |
| **Original predictors** | | | |
| *High frequency band (0.1-0.2 Hz)* | | | |
|  | dlPFC left | -0.004 | 0.001 |
|  | PMv right | 0.027 | 0.001 |
|  | PMv left | -0.052 | 0.001 |
|  | TPJ right | 0.273 | 0.001 |
|  | TPJ left | -0.095 | 0.001 |
| *Low frequency band (0.03-0.1 Hz)* | | | |
|  | dlPFC right | 0.111 | 0.001 |
|  | dlPFC left | 0.022 | 0.001 |
|  | PMv right | 0.019 | 0.001 |
|  | PMv left | -0.033 | 0.001 |
|  | TPJ right | -0.197 | 0.001 |
|  | TPJ left | 0.258 | 0.001 |
|  | SPL right | 0.099 | 0.001 |
| **Interaction predictors** | | | |
| *High frequency band (0.1-0.2 Hz)* | | | |
|  | dlPFC right x JA | 0.000 | 0.001 |
|  | dlPFC right x View Condition | 0.026 | 0.001 |
|  | PMv left x JA | 0.003 | 0.001 |
|  | PMv left x View Condition | -0.374 | 0.001 |
|  | TPJ right x JA | -0.003 | 0.001 |
|  | TPJ left x JA | 0.001 | 0.001 |
|  | TPJ left x View Condition | -0.211 | 0.001 |
|  | SPL left x JA | 0.001 | 0.001 |
| *Low frequency band (0.03-0.1 Hz)* | | | |
|  | dlPFC right x JA | 0.001 | 0.001 |
|  | dlPFC left x View Condition | 0.036 | 0.001 |
|  | PMv right x JA | 0.001 | 0.001 |
|  | PMv left x View Condition | 0.324 | 0.001 |
|  | TPJ right x JA | 0.002 | 0.001 |
|  | TPJ left x JA | -0.004 | 0.001 |
|  | TPJ left x View Condition | -0.094 | 0.001 |
|  | SPL left x JA | 0.001 | 0.001 |
|  | SPL left x View Condition | 0.190 | 0.001 |

Table S4. LASSO‐GLM survived predictors for the Full-view model.

This model included all regressors from the Big model plus Mutual Gaze as a regressor, run on the full-view dataset only. dlPFC: dorsolateral pre-frontal cortex; vPMC: ventral pre-motor cortex; TPJ: temporo-parietal junction; SPL: superior parietal lobe; JA: joint attention; MG: Mutual Gaze

|  | **Predictor** | **Coefficient** | **Lambda Used** |
| --- | --- | --- | --- |
| **Original predictors** | | | |
| *High frequency band (0.1-0.2 Hz)* | | | |
|  | dlPFC right | -0.023 | 0.001 |
|  | PMv left | 0.105 | 0.001 |
|  | TPJ right | 0.253 | 0.001 |
|  | TPJ left | 0.124 | 0.001 |
|  | SPL right | -0.048 | 0.001 |
|  | SPL left | 0.092 | 0.001 |
| *Low frequency band (0.03-0.1 Hz)* | | | |
|  | dlPFC right | 0.165 | 0.001 |
|  | dlPFC left | 0.080 | 0.001 |
|  | PMv right | -0.083 | 0.001 |
|  | PMv left | -0.018 | 0.001 |
|  | TPJ right | -0.251 | 0.001 |
|  | SPL right | 0.057 | 0.001 |
|  | SPL left | -0.024 | 0.001 |
| *Behaviour* | | | |
|  | MG | -0.009 | 0.001 |
| **Interaction predictors** | | | |
| *High frequency band (0.1-0.2 Hz)* | | | |
|  | dlPFC left x JA | 0.002 | 0.001 |
|  | dlPFC left x MG | -0.014 | 0.001 |
|  | PMv right x MG | 0.015 | 0.001 |
|  | PMv left x JA | 0.002 | 0.001 |
|  | PMv left x MG | -0.014 | 0.001 |
|  | TPJ right x JA | -0.005 | 0.001 |
|  | TPJ right x MG | 0.030 | 0.001 |
|  | TPJ left x MG | -0.008 | 0.001 |
| *Low frequency band (0.03-0.1 Hz)* | | | |
|  | dlPFC right x JA | 0.001 | 0.001 |
|  | dlPFC left x JA | -0.001 | 0.001 |
|  | PMv left x JA | 0.000 | 0.001 |
|  | PMv left x MG | -0.004 | 0.001 |
|  | TPJ right x JA | 0.003 | 0.001 |
|  | TPJ right x MG | -0.007 | 0.001 |
|  | TPJ left x JA | -0.001 | 0.001 |
|  | TPJ left x MG | 0.016 | 0.001 |
|  | SPL right x MG | 0.010 | 0.001 |

Table S5. LASSO‐GLM survived predictors for the Part-view model.

This model was analogous to the Big model, run on the part-view dataset only. dlPFC: dorsolateral pre-frontal cortex; vPMC: ventral pre-motor cortex; TPJ: temporo-parietal junction; SPL: superior parietal lobe; JA: joint attention.

|  | **Predictor** | **Coefficient** | **Lambda Used** |
| --- | --- | --- | --- |
| **Original predictors** | | | |
| *High frequency band (0.1-0.2 Hz)* | | | |
|  | dlPFC left | 0.094 | 0.001 |
|  | PMv right | 0.034 | 0.001 |
|  | PMv left | -0.283 | 0.001 |
|  | TPJ right | 0.278 | 0.001 |
|  | TPJ left | -0.292 | 0.001 |
|  | SPL right | -0.054 | 0.001 |
|  | SPL left | -0.133 | 0.001 |
| *Low frequency band (0.03-0.1 Hz)* | | | |
|  | dlPFC right | 0.024 | 0.001 |
|  | PMv right | 0.272 | 0.001 |
|  | PMv left | 0.246 | 0.001 |
|  | TPJ left | 0.176 | 0.001 |
|  | SPL left | 0.248 | 0.001 |
| *Behaviour* | | | |
|  | JA | 0.002 | 0.001 |
| **Interaction predictors** | | | |
| *High frequency band (0.1-0.2 Hz)* | | | |
|  | PMv right x JA | -0.001 | 0.001 |
|  | PMv left x JA | 0.001 | 0.001 |
|  | TPJ right x JA | -0.003 | 0.001 |
|  | TPJ left x JA | 0.002 | 0.001 |
|  | SPL left x JA | 0.003 | 0.001 |
| *Low frequency band (0.03-0.1 Hz)* | | | |
|  | dlPFC right x JA | 0.000 | 0.001 |
|  | dlPFC left x JA | 0.001 | 0.001 |
|  | PMv right x JA | -0.004 | 0.001 |
|  | TPJ right x JA | 0.000 | 0.001 |
|  | TPJ left x JA | -0.005 | 0.001 |
|  | SPL right x JA | 0.000 | 0.001 |

Table S6. xGLM results: beta contrasts for right dlPFC model.

|  | **ROI** | **tStat** | **p-value** | **p- value  (FDR corrected)** | **df** |
| --- | --- | --- | --- | --- | --- |
| **Breathing self > other** | | | | | |
|  | dlPFC right | 1.03 | 0.31 | 0.73 | 27 |
|  | dlPFC left | 1.83 | 0.08 | 0.62 | 29 |
|  | PMv right | -0.92 | 0.37 | 0.73 | 31 |
|  | PMv left | -0.57 | 0.57 | 0.80 | 31 |
|  | TPJ right | 0.53 | 0.60 | 0.80 | 31 |
|  | TPJ left | -0.25 | 0.80 | 0.92 | 31 |
|  | SPL right | 1.14 | 0.26 | 0.73 | 28 |
|  | SPL left | -0.08 | 0.94 | 0.94 | 28 |
| **Part- > Full-view** | | | | | |
|  | dlPFC right | 0.85 | 0.40 | 0.58 | 20 |
|  | dlPFC left | 1.12 | 0.27 | 0.55 | 20 |
|  | PMv right | 0.80 | 0.43 | 0.58 | 20 |
|  | PMv left | -1.34 | 0.19 | 0.55 | 20 |
|  | TPJ right | -0.30 | 0.77 | 0.77 | 20 |
|  | TPJ left | 1.27 | 0.22 | 0.55 | 20 |
|  | SPL right | -1.32 | 0.20 | 0.55 | 20 |
|  | SPL left | -0.35 | 0.73 | 0.77 | 20 |
| **Teacher > Learner** | | | | | |
|  | dlPFC right | 0.48 | 0.63 | 0.79 | 20 |
|  | dlPFC left | -1.75 | 0.10 | 0.38 | 20 |
|  | PMv right | 0.56 | 0.58 | 0.79 | 20 |
|  | PMv left | -1.08 | 0.29 | 0.58 | 20 |
|  | TPJ right | 1.23 | 0.23 | 0.58 | 20 |
|  | TPJ left | 0.41 | 0.69 | 0.79 | 20 |
|  | SPL right | 0.03 | 0.97 | 0.97 | 20 |
|  | SPL left | -1.87 | 0.08 | 0.38 | 20 |
| **JA** | | | | | |
|  | dlPFC right | 0.06 | 0.95 | 0.95 | 29 |
|  | dlPFC left | 1.26 | 0.22 | 0.86 | 33 |
|  | PMv right | -0.92 | 0.36 | 0.95 | 34 |
|  | PMv left | -0.14 | 0.89 | 0.95 | 35 |
|  | TPJ right | 0.57 | 0.57 | 0.95 | 35 |
|  | TPJ left | 0.29 | 0.77 | 0.95 | 35 |
|  | SPL right | -1.55 | 0.13 | 0.86 | 32 |
|  | SPL left | -0.50 | 0.62 | 0.95 | 31 |
| **Nodding self > other** | | | | | |
|  | dlPFC right | -0.25 | 0.80 | 0.92 | 29 |
|  | dlPFC left | -0.76 | 0.45 | 0.91 | 33 |
|  | PMv right | 1.59 | 0.12 | 0.64 | 34 |
|  | PMv left | -1.44 | 0.16 | 0.64 | 35 |
|  | TPJ right | 0.53 | 0.60 | 0.92 | 35 |
|  | TPJ left | 0.38 | 0.70 | 0.92 | 35 |
|  | SPL right | -0.79 | 0.44 | 0.91 | 32 |
|  | SPL left | -0.06 | 0.95 | 0.95 | 31 |
| **Speech self > other** | | | | | |
|  | dlPFC right | -1.46 | 0.15 | 0.31 | 29 |
|  | dlPFC left | 1.32 | 0.20 | 0.31 | 33 |
|  | PMv right | 0.07 | 0.95 | 0.95 | 34 |
|  | PMv left | -1.03 | 0.31 | 0.35 | 35 |
|  | TPJ right | 1.17 | 0.25 | 0.34 | 35 |
|  | TPJ left | 1.63 | 0.11 | 0.30 | 35 |
|  | SPL right | 2.62 | 0.01 | 0.11 | 32 |
|  | SPL left | 2.10 | 0.04 | 0.18 | 31 |

Table S7. xGLM results: beta contrasts for left dlPFC model.

|  | **ROI** | **tStat** | **p-value** | **p- value  (FDR corrected)** | **df** |
| --- | --- | --- | --- | --- | --- |
| **Breathing self > other** | | | | | |
|  | dlPFC right | 0.61 | 0.55 | 0.87 | 27 |
|  | dlPFC left | 1.76 | 0.09 | 0.71 | 29 |
|  | PMv right | -1.21 | 0.24 | 0.87 | 31 |
|  | PMv left | -0.60 | 0.55 | 0.87 | 31 |
|  | TPJ right | 0.35 | 0.73 | 0.87 | 31 |
|  | TPJ left | -0.16 | 0.87 | 0.87 | 31 |
|  | SPL right | 0.81 | 0.42 | 0.87 | 28 |
|  | SPL left | -0.25 | 0.80 | 0.87 | 28 |
| **Part- > Full-view** | | | | | |
|  | dlPFC right | 1.28 | 0.22 | 0.58 | 21 |
|  | dlPFC left | 0.10 | 0.92 | 0.96 | 21 |
|  | PMv right | -1.40 | 0.18 | 0.58 | 21 |
|  | PMv left | -0.27 | 0.79 | 0.96 | 21 |
|  | TPJ right | 1.76 | 0.09 | 0.58 | 21 |
|  | TPJ left | -0.82 | 0.42 | 0.84 | 21 |
|  | SPL right | 0.05 | 0.96 | 0.96 | 21 |
|  | SPL left | 0.29 | 0.78 | 0.96 | 21 |
| **Teacher > Learner** | | | | | |
|  | dlPFC right | 1.58 | 0.13 | 0.51 | 21 |
|  | dlPFC left | -0.46 | 0.65 | 0.74 | 21 |
|  | PMv right | 1.18 | 0.25 | 0.51 | 21 |
|  | PMv left | 0.10 | 0.92 | 0.92 | 21 |
|  | TPJ right | 0.76 | 0.45 | 0.67 | 21 |
|  | TPJ left | 0.68 | 0.50 | 0.67 | 21 |
|  | SPL right | 1.46 | 0.16 | 0.51 | 21 |
|  | SPL left | -1.28 | 0.21 | 0.51 | 21 |
| **JA** | | | | | |
|  | dlPFC right | 0.11 | 0.92 | 0.97 | 29 |
|  | dlPFC left | 1.65 | 0.11 | 0.87 | 33 |
|  | PMv right | -0.75 | 0.46 | 0.94 | 34 |
|  | PMv left | 0.04 | 0.97 | 0.97 | 35 |
|  | TPJ right | 0.66 | 0.51 | 0.94 | 35 |
|  | TPJ left | 0.42 | 0.68 | 0.94 | 35 |
|  | SPL right | -1.17 | 0.25 | 0.94 | 32 |
|  | SPL left | -0.38 | 0.71 | 0.94 | 31 |
| **Nodding self > other** | | | | | |
|  | dlPFC right | -0.30 | 0.77 | 0.91 | 29 |
|  | dlPFC left | -0.77 | 0.45 | 0.90 | 33 |
|  | PMv right | 1.51 | 0.14 | 0.82 | 34 |
|  | PMv left | -1.03 | 0.31 | 0.82 | 35 |
|  | TPJ right | 0.43 | 0.67 | 0.91 | 35 |
|  | TPJ left | 0.26 | 0.79 | 0.91 | 35 |
|  | SPL right | -1.10 | 0.28 | 0.82 | 32 |
|  | SPL left | 0.02 | 0.98 | 0.98 | 31 |
| **Speech self > other** | | | | | |
|  | dlPFC right | -1.36 | 0.18 | 0.29 | 29 |
|  | dlPFC left | 1.87 | 0.07 | 0.14 | 33 |
|  | PMv right | 0.22 | 0.82 | 0.82 | 34 |
|  | PMv left | -0.47 | 0.64 | 0.74 | 35 |
|  | TPJ right | 1.13 | 0.27 | 0.35 | 35 |
|  | TPJ left | 1.97 | 0.06 | 0.14 | 35 |
|  | SPL right | 2.73 | 0.01 | 0.08 | 32 |
|  | SPL left | 2.45 | 0.02 | 0.08 | 31 |

Table S8. xGLM results: beta contrasts for right PMv model.

|  | **ROI** | **tStat** | **p-value** | **p- value  (FDR corrected)** | **df** |
| --- | --- | --- | --- | --- | --- |
| **Breathing self > other** | | | | | |
|  | dlPFC right | 0.99 | 0.33 | 0.78 | 27 |
|  | dlPFC left | 2.17 | 0.04 | 0.31 | 29 |
|  | PMv right | -0.88 | 0.39 | 0.78 | 31 |
|  | PMv left | -0.22 | 0.83 | 0.95 | 31 |
|  | TPJ right | 0.32 | 0.75 | 0.95 | 31 |
|  | TPJ left | -0.06 | 0.95 | 0.95 | 31 |
|  | SPL right | 0.87 | 0.39 | 0.78 | 28 |
|  | SPL left | 0.07 | 0.95 | 0.95 | 28 |
| **Part- > Full-view** | | | | | |
|  | dlPFC right | 0.12 | 0.91 | 0.96 | 23 |
|  | dlPFC left | -1.44 | 0.16 | 0.44 | 23 |
|  | PMv right | 0.50 | 0.62 | 0.96 | 23 |
|  | PMv left | -0.87 | 0.39 | 0.79 | 23 |
|  | TPJ right | -0.05 | 0.96 | 0.96 | 23 |
|  | TPJ left | -2.52 | 0.02 | 0.15 | 23 |
|  | SPL right | -1.69 | 0.10 | 0.42 | 23 |
|  | SPL left | -0.27 | 0.79 | 0.96 | 23 |
| **Teacher > Learner** | | | | | |
|  | dlPFC right | 0.67 | 0.51 | 0.68 | 23 |
|  | dlPFC left | -0.58 | 0.57 | 0.68 | 23 |
|  | PMv right | 0.71 | 0.49 | 0.68 | 23 |
|  | PMv left | -0.58 | 0.57 | 0.68 | 23 |
|  | TPJ right | -0.03 | 0.98 | 0.98 | 23 |
|  | TPJ left | 0.62 | 0.54 | 0.68 | 23 |
|  | SPL right | 0.53 | 0.60 | 0.68 | 23 |
|  | SPL left | -0.81 | 0.43 | 0.68 | 23 |
| **JA** | | | | | |
|  | dlPFC right | 0.19 | 0.85 | 0.91 | 29 |
|  | dlPFC left | 1.62 | 0.11 | 0.73 | 33 |
|  | PMv right | -1.11 | 0.27 | 0.73 | 34 |
|  | PMv left | -0.12 | 0.91 | 0.91 | 35 |
|  | TPJ right | 0.46 | 0.65 | 0.91 | 35 |
|  | TPJ left | 0.29 | 0.77 | 0.91 | 35 |
|  | SPL right | -1.26 | 0.22 | 0.73 | 32 |
|  | SPL left | -0.36 | 0.72 | 0.91 | 31 |
| **Nodding self > other** | | | | | |
|  | dlPFC right | -0.26 | 0.80 | 0.91 | 29 |
|  | dlPFC left | -0.92 | 0.36 | 0.72 | 33 |
|  | PMv right | 1.48 | 0.15 | 0.72 | 34 |
|  | PMv left | -1.11 | 0.28 | 0.72 | 35 |
|  | TPJ right | 0.48 | 0.63 | 0.91 | 35 |
|  | TPJ left | 0.07 | 0.95 | 0.95 | 35 |
|  | SPL right | -0.93 | 0.36 | 0.72 | 32 |
|  | SPL left | 0.40 | 0.69 | 0.91 | 31 |
| **Speech self > other** | | | | | |
|  | dlPFC right | -1.21 | 0.24 | 0.32 | 29 |
|  | dlPFC left | 1.32 | 0.20 | 0.32 | 33 |
|  | PMv right | 0.01 | 0.99 | 0.99 | 34 |
|  | PMv left | -0.39 | 0.70 | 0.80 | 35 |
|  | TPJ right | 1.28 | 0.21 | 0.32 | 35 |
|  | TPJ left | 1.85 | 0.07 | 0.19 | 35 |
|  | SPL right | 3.03 | 0.00 | 0.04 | 32 |
|  | SPL left | 2.61 | 0.01 | 0.06 | 31 |

Table S9. xGLM results: beta contrasts for left PMv model.

|  | **ROI** | **tStat** | **p-value** | **p- value  (FDR corrected)** | **df** |
| --- | --- | --- | --- | --- | --- |
| **Breathing self > other** | | | | | |
|  | dlPFC right | 1.03 | 0.31 | 0.77 | 27 |
|  | dlPFC left | 1.95 | 0.06 | 0.49 | 29 |
|  | PMv right | -0.88 | 0.38 | 0.77 | 31 |
|  | PMv left | -0.23 | 0.82 | 0.97 | 31 |
|  | TPJ right | 0.48 | 0.64 | 0.97 | 31 |
|  | TPJ left | -0.04 | 0.97 | 0.97 | 31 |
|  | SPL right | 1.05 | 0.30 | 0.77 | 28 |
|  | SPL left | 0.05 | 0.96 | 0.97 | 28 |
| **Part- > Full-view** | | | | | |
|  | dlPFC right | -1.21 | 0.24 | 0.50 | 23 |
|  | dlPFC left | -1.34 | 0.19 | 0.50 | 23 |
|  | PMv right | -1.07 | 0.30 | 0.50 | 23 |
|  | PMv left | -2.51 | 0.02 | 0.16 | 23 |
|  | TPJ right | -0.90 | 0.38 | 0.50 | 23 |
|  | TPJ left | -0.51 | 0.61 | 0.70 | 23 |
|  | SPL right | -1.01 | 0.32 | 0.50 | 23 |
|  | SPL left | 0.33 | 0.74 | 0.74 | 23 |
| **Teacher > Learner** | | | | | |
|  | dlPFC right | 0.60 | 0.55 | 0.88 | 23 |
|  | dlPFC left | -0.16 | 0.88 | 0.88 | 23 |
|  | PMv right | 1.30 | 0.20 | 0.82 | 23 |
|  | PMv left | -0.16 | 0.88 | 0.88 | 23 |
|  | TPJ right | -0.37 | 0.72 | 0.88 | 23 |
|  | TPJ left | 1.34 | 0.19 | 0.82 | 23 |
|  | SPL right | 0.37 | 0.71 | 0.88 | 23 |
|  | SPL left | -0.96 | 0.35 | 0.88 | 23 |
| **JA** | | | | | |
|  | dlPFC right | 0.15 | 0.88 | 0.90 | 29 |
|  | dlPFC left | 1.76 | 0.09 | 0.70 | 33 |
|  | PMv right | -0.92 | 0.37 | 0.90 | 34 |
|  | PMv left | 0.13 | 0.90 | 0.90 | 35 |
|  | TPJ right | 0.72 | 0.48 | 0.90 | 35 |
|  | TPJ left | 0.34 | 0.74 | 0.90 | 35 |
|  | SPL right | -1.31 | 0.20 | 0.80 | 32 |
|  | SPL left | -0.19 | 0.85 | 0.90 | 31 |
| **Nodding self > other** | | | | | |
|  | dlPFC right | -0.14 | 0.89 | 0.89 | 29 |
|  | dlPFC left | -0.81 | 0.42 | 0.68 | 33 |
|  | PMv right | 1.55 | 0.13 | 0.68 | 34 |
|  | PMv left | -1.27 | 0.21 | 0.68 | 35 |
|  | TPJ right | 0.54 | 0.59 | 0.68 | 35 |
|  | TPJ left | 0.63 | 0.53 | 0.68 | 35 |
|  | SPL right | -0.94 | 0.36 | 0.68 | 32 |
|  | SPL left | 0.57 | 0.57 | 0.68 | 31 |
| **Speech self > other** | | | | | |
|  | dlPFC right | -1.40 | 0.17 | 0.28 | 29 |
|  | dlPFC left | 2.05 | 0.05 | 0.10 | 33 |
|  | PMv right | 0.20 | 0.85 | 0.89 | 34 |
|  | PMv left | -0.14 | 0.89 | 0.89 | 35 |
|  | TPJ right | 1.28 | 0.21 | 0.28 | 35 |
|  | TPJ left | 2.25 | 0.03 | 0.08 | 35 |
|  | SPL right | 2.82 | 0.01 | 0.03 | 32 |
|  | SPL left | 2.95 | 0.01 | 0.03 | 31 |

Table S10. xGLM results: beta contrasts for right TPJ model.

|  | **ROI** | **tStat** | **p-value** | **p- value  (FDR corrected)** | **df** |
| --- | --- | --- | --- | --- | --- |
| **Breathing self > other** | | | | | |
|  | dlPFC right | 0.90 | 0.37 | 0.79 | 27 |
|  | dlPFC left | 2.05 | 0.05 | 0.40 | 29 |
|  | PMv right | -0.94 | 0.35 | 0.79 | 31 |
|  | PMv left | -0.55 | 0.59 | 0.88 | 31 |
|  | TPJ right | 0.45 | 0.66 | 0.88 | 31 |
|  | TPJ left | -0.06 | 0.95 | 0.96 | 31 |
|  | SPL right | 0.87 | 0.39 | 0.79 | 28 |
|  | SPL left | 0.05 | 0.96 | 0.96 | 28 |
| **Part- > Full-view** | | | | | |
|  | dlPFC right | -0.34 | 0.74 | 0.85 | 23 |
|  | dlPFC left | 0.57 | 0.58 | 0.85 | 23 |
|  | PMv right | -0.38 | 0.71 | 0.85 | 23 |
|  | PMv left | 0.02 | 0.98 | 0.98 | 23 |
|  | TPJ right | 0.49 | 0.63 | 0.85 | 23 |
|  | TPJ left | -0.71 | 0.49 | 0.85 | 23 |
|  | SPL right | 1.79 | 0.09 | 0.46 | 23 |
|  | SPL left | 1.64 | 0.11 | 0.46 | 23 |
| **Teacher > Learner** | | | | | |
|  | dlPFC right | 0.30 | 0.77 | 0.88 | 23 |
|  | dlPFC left | -0.66 | 0.52 | 0.88 | 23 |
|  | PMv right | -0.89 | 0.38 | 0.88 | 23 |
|  | PMv left | 0.34 | 0.74 | 0.88 | 23 |
|  | TPJ right | -0.13 | 0.90 | 0.90 | 23 |
|  | TPJ left | 1.05 | 0.30 | 0.88 | 23 |
|  | SPL right | 0.68 | 0.50 | 0.88 | 23 |
|  | SPL left | 0.44 | 0.66 | 0.88 | 23 |
| **JA** | | | | | |
|  | dlPFC right | 0.11 | 0.92 | 0.99 | 29 |
|  | dlPFC left | 1.47 | 0.15 | 0.60 | 33 |
|  | PMv right | -0.94 | 0.35 | 0.86 | 34 |
|  | PMv left | -0.02 | 0.99 | 0.99 | 35 |
|  | TPJ right | 0.80 | 0.43 | 0.86 | 35 |
|  | TPJ left | 0.26 | 0.80 | 0.99 | 35 |
|  | SPL right | -1.49 | 0.15 | 0.60 | 32 |
|  | SPL left | -0.54 | 0.60 | 0.95 | 31 |
| **Nodding self > other** | | | | | |
|  | dlPFC right | -0.28 | 0.78 | 0.80 | 29 |
|  | dlPFC left | -0.86 | 0.40 | 0.80 | 33 |
|  | PMv right | 1.52 | 0.14 | 0.80 | 34 |
|  | PMv left | -1.25 | 0.22 | 0.80 | 35 |
|  | TPJ right | 0.32 | 0.75 | 0.80 | 35 |
|  | TPJ left | 0.31 | 0.76 | 0.80 | 35 |
|  | SPL right | -0.82 | 0.42 | 0.80 | 32 |
|  | SPL left | 0.25 | 0.80 | 0.80 | 31 |
| **Speech self > other** | | | | | |
|  | dlPFC right | -1.13 | 0.27 | 0.41 | 29 |
|  | dlPFC left | 1.74 | 0.09 | 0.18 | 33 |
|  | PMv right | 0.07 | 0.94 | 0.94 | 34 |
|  | PMv left | -0.37 | 0.72 | 0.82 | 35 |
|  | TPJ right | 1.04 | 0.31 | 0.41 | 35 |
|  | TPJ left | 2.35 | 0.02 | 0.07 | 35 |
|  | SPL right | 3.05 | 0.00 | 0.04 | 32 |
|  | SPL left | 2.33 | 0.03 | 0.07 | 31 |

Table S11. xGLM results: beta contrasts for left TPJ model.

|  | **ROI** | **tStat** | **p-value** | **p- value  (FDR corrected)** | **df** |
| --- | --- | --- | --- | --- | --- |
| **Breathing self > other** | | | | | |
|  | dlPFC right | 0.80 | 0.43 | 0.92 | 27 |
|  | dlPFC left | 1.89 | 0.07 | 0.55 | 29 |
|  | PMv right | -1.25 | 0.22 | 0.88 | 31 |
|  | PMv left | -0.41 | 0.69 | 0.92 | 31 |
|  | TPJ right | 0.52 | 0.61 | 0.92 | 31 |
|  | TPJ left | 0.15 | 0.88 | 0.98 | 31 |
|  | SPL right | 0.64 | 0.53 | 0.92 | 28 |
|  | SPL left | 0.02 | 0.98 | 0.98 | 28 |
| **Part- > Full-view** | | | | | |
|  | dlPFC right | 0.32 | 0.75 | 0.86 | 23 |
|  | dlPFC left | -1.22 | 0.24 | 0.63 | 23 |
|  | PMv right | -2.19 | 0.04 | 0.31 | 23 |
|  | PMv left | -1.60 | 0.12 | 0.49 | 23 |
|  | TPJ right | -0.72 | 0.48 | 0.64 | 23 |
|  | TPJ left | -0.75 | 0.46 | 0.64 | 23 |
|  | SPL right | 0.79 | 0.44 | 0.64 | 23 |
|  | SPL left | 0.17 | 0.86 | 0.86 | 23 |
| **Teacher > Learner** | | | | | |
|  | dlPFC right | 0.32 | 0.75 | 0.86 | 23 |
|  | dlPFC left | -0.66 | 0.52 | 0.86 | 23 |
|  | PMv right | 0.12 | 0.91 | 0.91 | 23 |
|  | PMv left | -1.15 | 0.26 | 0.86 | 23 |
|  | TPJ right | -0.48 | 0.64 | 0.86 | 23 |
|  | TPJ left | -0.38 | 0.71 | 0.86 | 23 |
|  | SPL right | -0.44 | 0.67 | 0.86 | 23 |
|  | SPL left | -1.71 | 0.10 | 0.81 | 23 |
| **JA** | | | | | |
|  | dlPFC right | 0.12 | 0.90 | 0.90 | 29 |
|  | dlPFC left | 1.63 | 0.11 | 0.56 | 33 |
|  | PMv right | -0.85 | 0.40 | 0.90 | 34 |
|  | PMv left | 0.14 | 0.89 | 0.90 | 35 |
|  | TPJ right | 0.70 | 0.49 | 0.90 | 35 |
|  | TPJ left | 0.35 | 0.73 | 0.90 | 35 |
|  | SPL right | -1.51 | 0.14 | 0.56 | 32 |
|  | SPL left | -0.42 | 0.68 | 0.90 | 31 |
| **Nodding self > other** | | | | | |
|  | dlPFC right | -0.38 | 0.71 | 0.81 | 29 |
|  | dlPFC left | -0.39 | 0.70 | 0.81 | 33 |
|  | PMv right | 1.60 | 0.12 | 0.81 | 34 |
|  | PMv left | -0.98 | 0.33 | 0.81 | 35 |
|  | TPJ right | 0.60 | 0.55 | 0.81 | 35 |
|  | TPJ left | 0.49 | 0.63 | 0.81 | 35 |
|  | SPL right | -0.67 | 0.51 | 0.81 | 32 |
|  | SPL left | 0.22 | 0.83 | 0.83 | 31 |
| **Speech self > other** | | | | | |
|  | dlPFC right | -1.35 | 0.19 | 0.30 | 29 |
|  | dlPFC left | 1.93 | 0.06 | 0.12 | 33 |
|  | PMv right | 0.29 | 0.78 | 0.78 | 34 |
|  | PMv left | -0.50 | 0.62 | 0.71 | 35 |
|  | TPJ right | 1.11 | 0.28 | 0.37 | 35 |
|  | TPJ left | 2.22 | 0.03 | 0.09 | 35 |
|  | SPL right | 2.62 | 0.01 | 0.06 | 32 |
|  | SPL left | 2.56 | 0.02 | 0.06 | 31 |

Table S12. xGLM results: beta contrasts for right SPL model.

|  | **ROI** | **tStat** | **p-value** | **p- value  (FDR corrected)** | **df** |
| --- | --- | --- | --- | --- | --- |
| **Breathing self > other** | | | | | |
|  | dlPFC right | 0.71 | 0.48 | 0.95 | 27 |
|  | dlPFC left | 2.01 | 0.05 | 0.43 | 29 |
|  | PMv right | -0.92 | 0.37 | 0.95 | 31 |
|  | PMv left | -0.28 | 0.78 | 0.95 | 31 |
|  | TPJ right | 0.53 | 0.60 | 0.95 | 31 |
|  | TPJ left | -0.18 | 0.85 | 0.95 | 31 |
|  | SPL right | 0.95 | 0.35 | 0.95 | 28 |
|  | SPL left | 0.06 | 0.95 | 0.95 | 28 |
| **Part- > Full-view** | | | | | |
|  | dlPFC right | -0.06 | 0.95 | 0.95 | 22 |
|  | dlPFC left | -0.84 | 0.41 | 0.52 | 22 |
|  | PMv right | 0.85 | 0.40 | 0.52 | 22 |
|  | PMv left | -1.60 | 0.12 | 0.48 | 22 |
|  | TPJ right | 0.76 | 0.45 | 0.52 | 22 |
|  | TPJ left | 1.38 | 0.18 | 0.48 | 22 |
|  | SPL right | 2.63 | 0.02 | 0.12 | 22 |
|  | SPL left | 1.16 | 0.26 | 0.52 | 22 |
| **Teacher > Learner** | | | | | |
|  | dlPFC right | -0.83 | 0.42 | 0.70 | 22 |
|  | dlPFC left | -1.05 | 0.31 | 0.70 | 22 |
|  | PMv right | 1.24 | 0.23 | 0.70 | 22 |
|  | PMv left | 0.64 | 0.53 | 0.70 | 22 |
|  | TPJ right | 1.51 | 0.14 | 0.70 | 22 |
|  | TPJ left | 0.30 | 0.76 | 0.87 | 22 |
|  | SPL right | -0.16 | 0.87 | 0.87 | 22 |
|  | SPL left | -0.72 | 0.48 | 0.70 | 22 |
| **JA** | | | | | |
|  | dlPFC right | 0.13 | 0.90 | 0.91 | 29 |
|  | dlPFC left | 1.56 | 0.13 | 0.90 | 33 |
|  | PMv right | -0.79 | 0.44 | 0.91 | 34 |
|  | PMv left | 0.12 | 0.91 | 0.91 | 35 |
|  | TPJ right | 0.60 | 0.56 | 0.91 | 35 |
|  | TPJ left | 0.25 | 0.81 | 0.91 | 35 |
|  | SPL right | -1.24 | 0.22 | 0.90 | 32 |
|  | SPL left | -0.53 | 0.60 | 0.91 | 31 |
| **Nodding self > other** | | | | | |
|  | dlPFC right | -0.43 | 0.67 | 0.89 | 29 |
|  | dlPFC left | -0.92 | 0.36 | 0.73 | 33 |
|  | PMv right | 1.48 | 0.15 | 0.59 | 34 |
|  | PMv left | -1.22 | 0.23 | 0.62 | 35 |
|  | TPJ right | 0.59 | 0.56 | 0.89 | 35 |
|  | TPJ left | 0.20 | 0.85 | 0.91 | 35 |
|  | SPL right | -1.60 | 0.12 | 0.59 | 32 |
|  | SPL left | 0.11 | 0.91 | 0.91 | 31 |
| **Speech self > other** | | | | | |
|  | dlPFC right | -0.98 | 0.33 | 0.45 | 29 |
|  | dlPFC left | 1.94 | 0.06 | 0.12 | 33 |
|  | PMv right | -0.01 | 0.99 | 0.99 | 34 |
|  | PMv left | -0.62 | 0.54 | 0.62 | 35 |
|  | TPJ right | 1.18 | 0.25 | 0.39 | 35 |
|  | TPJ left | 2.20 | 0.03 | 0.09 | 35 |
|  | SPL right | 2.95 | 0.01 | 0.05 | 32 |
|  | SPL left | 2.27 | 0.03 | 0.09 | 31 |

Table S13. xGLM results: beta contrasts for left SPL model.

|  | **ROI** | **tStat** | **p-value** | **p- value  (FDR corrected)** | **df** |
| --- | --- | --- | --- | --- | --- |
| **Breathing self > other** | | | | | |
|  | dlPFC right | 0.80 | 0.43 | 0.86 | 27 |
|  | dlPFC left | 1.99 | 0.06 | 0.45 | 29 |
|  | PMv right | -0.87 | 0.39 | 0.86 | 31 |
|  | PMv left | -0.47 | 0.64 | 0.86 | 31 |
|  | TPJ right | 0.55 | 0.59 | 0.86 | 31 |
|  | TPJ left | -0.20 | 0.84 | 0.93 | 31 |
|  | SPL right | 1.03 | 0.31 | 0.86 | 28 |
|  | SPL left | 0.08 | 0.93 | 0.93 | 28 |
| **Part- > Full-view** | | | | | |
|  | dlPFC right | -0.09 | 0.93 | 0.97 | 22 |
|  | dlPFC left | -0.25 | 0.81 | 0.97 | 22 |
|  | PMv right | 1.26 | 0.22 | 0.49 | 22 |
|  | PMv left | -0.04 | 0.97 | 0.97 | 22 |
|  | TPJ right | 1.75 | 0.09 | 0.49 | 22 |
|  | TPJ left | 0.22 | 0.83 | 0.97 | 22 |
|  | SPL right | 1.20 | 0.24 | 0.49 | 22 |
|  | SPL left | 1.54 | 0.14 | 0.49 | 22 |
| **Teacher > Learner** | | | | | |
|  | dlPFC right | 1.93 | 0.07 | 0.21 | 22 |
|  | dlPFC left | 1.74 | 0.10 | 0.21 | 22 |
|  | PMv right | 1.70 | 0.10 | 0.21 | 22 |
|  | PMv left | 3.18 | 0.00 | 0.03 | 22 |
|  | TPJ right | 1.07 | 0.30 | 0.34 | 22 |
|  | TPJ left | 0.81 | 0.43 | 0.43 | 22 |
|  | SPL right | 1.48 | 0.15 | 0.23 | 22 |
|  | SPL left | 1.41 | 0.17 | 0.23 | 22 |
| **JA** | | | | | |
|  | dlPFC right | 0.10 | 0.92 | 0.92 | 29 |
|  | dlPFC left | 1.48 | 0.15 | 0.87 | 33 |
|  | PMv right | -0.84 | 0.40 | 0.90 | 34 |
|  | PMv left | 0.34 | 0.74 | 0.90 | 35 |
|  | TPJ right | 0.70 | 0.49 | 0.90 | 35 |
|  | TPJ left | 0.48 | 0.64 | 0.90 | 35 |
|  | SPL right | -1.26 | 0.22 | 0.87 | 32 |
|  | SPL left | -0.27 | 0.79 | 0.90 | 31 |
| **Nodding self > other** | | | | | |
|  | dlPFC right | 0.05 | 0.96 | 0.96 | 29 |
|  | dlPFC left | -0.73 | 0.47 | 0.94 | 33 |
|  | PMv right | 1.71 | 0.10 | 0.78 | 34 |
|  | PMv left | -1.10 | 0.28 | 0.94 | 35 |
|  | TPJ right | 0.54 | 0.59 | 0.94 | 35 |
|  | TPJ left | 0.22 | 0.83 | 0.94 | 35 |
|  | SPL right | -0.78 | 0.44 | 0.94 | 32 |
|  | SPL left | 0.31 | 0.76 | 0.94 | 31 |
| **Speech self > other** | | | | | |
|  | dlPFC right | -1.12 | 0.27 | 0.36 | 29 |
|  | dlPFC left | 2.06 | 0.05 | 0.09 | 33 |
|  | PMv right | 0.18 | 0.86 | 0.86 | 34 |
|  | PMv left | -0.39 | 0.70 | 0.80 | 35 |
|  | TPJ right | 1.32 | 0.20 | 0.31 | 35 |
|  | TPJ left | 2.24 | 0.03 | 0.08 | 35 |
|  | SPL right | 3.06 | 0.00 | 0.02 | 32 |
|  | SPL left | 2.94 | 0.01 | 0.02 | 31 |


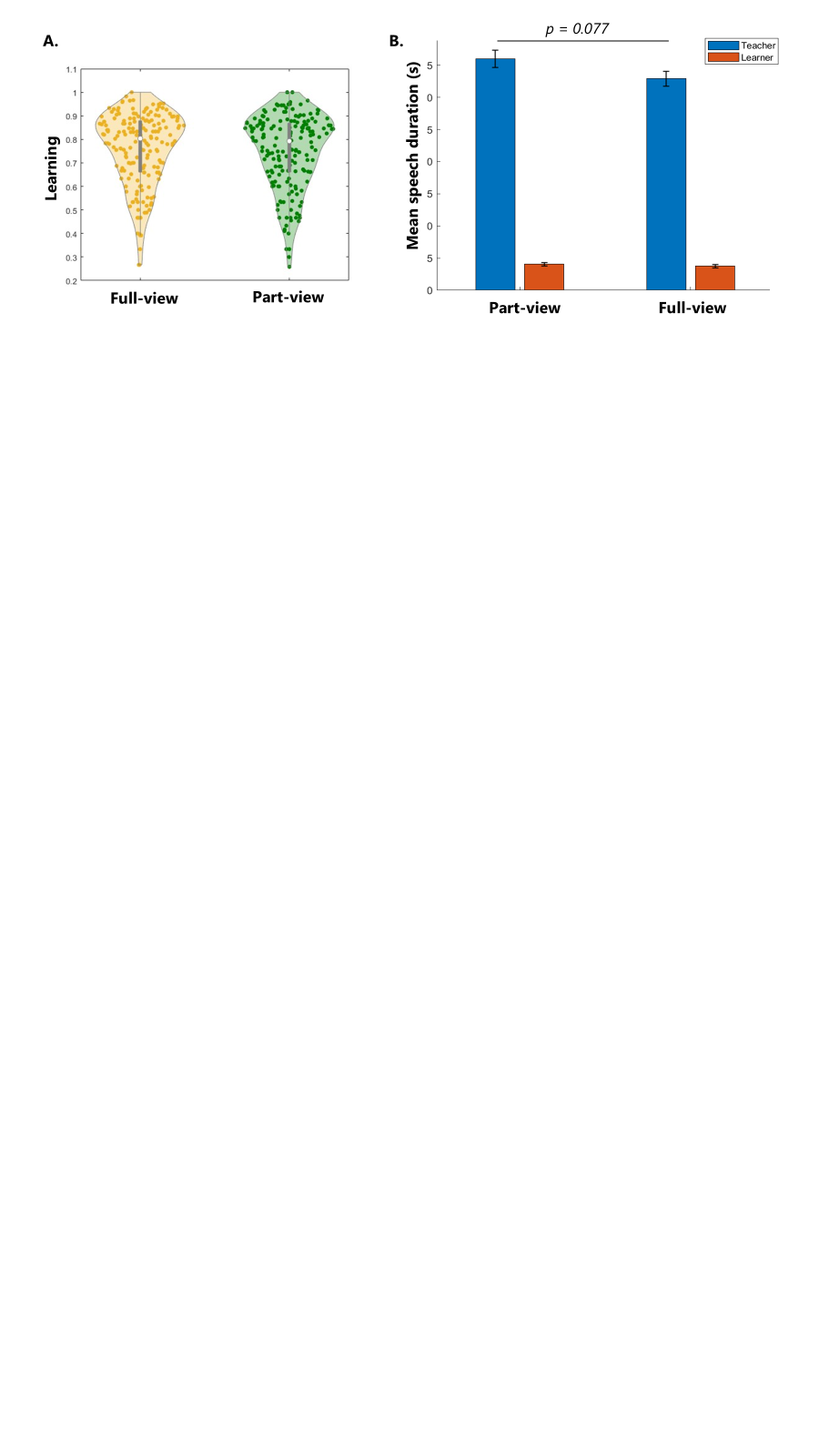


Figure S1. A. Learning performance by condition. B. Mean speech duration for Learner and Teacher role by condition.
